# SARS-CoV-2 booster vaccination rescues attenuated IgG1 memory B cell response in primary antibody deficiency patients

**DOI:** 10.3389/fimmu.2022.1033770

**Published:** 2022-12-22

**Authors:** Frank J. Lin, Alexa Michelle Altman Doss, Hannah G. Davis-Adams, Lucas J. Adams, Christopher H. Hanson, Laura A. VanBlargan, Chieh-Yu Liang, Rita E. Chen, Jennifer Marie Monroy, H. James Wedner, Anthony Kulczycki, Tarisa L. Mantia, Caitlin C. O’Shaughnessy, Saravanan Raju, Fang R. Zhao, Elise Rizzi, Christopher J. Rigell, Tiffany Biason Dy, Andrew L. Kau, Zhen Ren, Jackson S. Turner, Jane A. O’Halloran, Rachel M. Presti, Daved H. Fremont, Peggy L. Kendall, Ali H. Ellebedy, Philip A. Mudd, Michael S. Diamond, Ofer Zimmerman, Brian J. Laidlaw

**Affiliations:** ^1^ Department of Medicine, Washington University School of Medicine, St. Louis, MO, United States; ^2^ Department of Pediatrics, Washington University School of Medicine, St. Louis, MO, United States; ^3^ Department of Pathology and Immunology, Washington University School of Medicine, St. Louis, MO, United States; ^4^ Center for Women’s Infectious Disease Research, Washington University School of Medicine, St. Louis, MO, United States; ^5^ Center for Vaccines and Immunity to Microbial Pathogens, Washington University School of Medicine, Saint Louis, MO, United States; ^6^ The Andrew M. and Jane M. Bursky Center for Human Immunology & Immunotherapy Programs, Washington University School of Medicine, Saint Louis, MO, United States; ^7^ Department of Emergency Medicine, Washington University School of Medicine, St. Louis, MO, United States; ^8^ Department of Molecular Microbiology, Washington University School of Medicine, St. Louis, MO, United States

**Keywords:** SARS-CoV-2, immune memory, B cells, vaccination, primary antibody deficiency, common variable immunodeficiency, hypogammaglobulinemia, specific antibody deficiency

## Abstract

**Background:**

Although SARS-CoV-2 vaccines have proven effective in eliciting a protective immune response in healthy individuals, their ability to induce a durable immune response in immunocompromised individuals remains poorly understood. Primary antibody deficiency (PAD) syndromes are among the most common primary immunodeficiency disorders in adults and are characterized by hypogammaglobulinemia and impaired ability to mount robust antibody responses following infection or vaccination.

**Methods:**

Here, we present an analysis of both the B and T cell response in a prospective cohort of 30 individuals with PAD up to 150 days following initial COVID-19 vaccination and 150 days post mRNA booster vaccination.

**Results:**

After the primary vaccination series, many of the individuals with PAD syndromes mounted SARS-CoV-2 specific memory B and CD4^+^ T cell responses that overall were comparable to healthy individuals. Nonetheless, individuals with PAD syndromes had reduced IgG1^+^ and CD11c^+^ memory B cell responses following the primary vaccination series, with the defect in IgG1 class-switching rescued following mRNA booster doses. Boosting also elicited an increase in the SARS-CoV-2-specific B and T cell response and the development of Omicron-specific memory B cells in COVID-19-naïve PAD patients. Individuals that lacked detectable B cell responses following primary vaccination did not benefit from booster vaccination.

**Conclusion:**

Together, these data indicate that SARS-CoV-2 vaccines elicit memory B and T cells in most PAD patients and highlights the importance of booster vaccination in immunodeficient individuals.

## Introduction

Severe acute respiratory syndrome coronavirus 2 (SARS-CoV-2) is the causative agent of COVID-19 and has infected more than 625 million individuals resulting in over 6.5 million deaths worldwide as of October 2022 (1). The mRNA-based Pfizer-BioNTech (BNT162b2) and Moderna (mRNA-1273), the vector-based Johnson & Johnson (Ad26.COV2.S), and the protein subunit-based Novavax (NVX-CoV2373) SARS-CoV-2 vaccines have either full or Emergency Use approval in the United States and have demonstrated efficacy in preventing symptomatic and asymptomatic infections (2–9). Although SARS-CoV-2-specific antibody titers wane over time, a durable cellular immune response is detectable for at least 6 months following completion of the primary vaccination series (10). The administration of an mRNA booster vaccination leads to a rapid increase in serum antibody titers that enables neutralization of viral variants, including Omicron (BA.1), which can evade immunity elicited by the primary vaccination series (11–14).

Individuals with medical conditions that compromise their ability to mount immune responses, such as primary and secondary immunodeficiencies, are at increased risk for severe illness and death following SARS-CoV-2 infection (15, 16). These individuals also have an impaired SARS-CoV-2-specific antibody response following a primary vaccination series (17–26). Accordingly, moderately or severely immunosuppressed patients are recommended by the Centers for Disease Control and Prevention (CDC) to receive a third dose as part of their primary series against SARS-CoV-2 with a fourth dose recommended 3 months later (27). Administration of booster doses leads to an enhanced SARS-CoV-2-specific antibody response in immunocompromised individuals (19, 26).

Primary antibody deficiency (PAD) syndromes are the most common symptomatic primary immunodeficiency in adults and are characterized by an impaired ability to mount an antibody response following infection or vaccination (28). The etiology of PAD syndromes is unknown in most patients, with only 25-35% of cases explained by inborn errors of immunity (29–33). Individuals with PAD syndromes are at increased risk of recurrent and severe infections, autoimmunity, allergic disease, and malignancies (28). Most individuals with PAD syndromes receive intravenous or subcutaneous immunoglobulin replacement therapy every 1 to 4 weeks to reduce the frequency and severity of infections (34). However, immunoglobulin replacement therapy consists of immunoglobulin donated up to one year earlier and is unlikely to contain high titers of neutralizing antibodies specific for the variant strain of SARS-CoV-2 that is dominant at the time of administration (26, 35, 36).

We previously found that COVID-19-naïve individuals with PAD syndromes had attenuated anti-SARS-CoV-2-spike and receptor binding domain (RBD) antibody responses following primary vaccination relative to healthy donors (26). The administration of a booster vaccine dose increased antibody titers, avidity, and neutralization activity against WA1/2020, Delta (B.1.617.2) and Omicron variants (26). However, SARS-CoV-2-specific total and neutralizing antibody titers waned by day 90 post-boost suggesting they may be insufficient to maintain long-term protective immunity in individuals with PAD syndromes (26). In the current study, we performed a prospective analysis of the SARS-CoV-2-specific B and T cell response following SARS-CoV-2 primary and booster vaccination in PAD patients. Unexpectedly, most individuals with PAD syndromes generated memory T and B cell responses that were comparable in magnitude to the response in healthy donors following the primary vaccination series, although we observed defects in the development of spike-specific IgG1^+^ and CD11c^+^ memory B cells. Administration of a booster dose led to a further enhancement in B and T cell responses, including the development of Omicron-specific B cells and rescue of the spike-specific IgG1^+^ memory B cell response. This study provides insight into the ability of mRNA vaccination and boosting to induce protective memory B and T cells responses in PAD patients.

## Materials and methods

### Primary antibody deficiency syndromes cohort

The study was approved by the Institutional Review Board of Washington University School of Medicine (Approval # 202104138). Patients were identified by a medical record search for PAD syndromes: common variable immune deficiency syndrome (CVID), hypogammaglobulinemia and specific antibody deficiency (SAD), and their records were reviewed to confirm their diagnosis and verify they met the inclusion criteria (see supplemental methods). COVID-19 vaccination status was reviewed, and subjects were contacted if they were within the vaccination window or not yet immunized. Inclusion criteria included males and females over 18 years of age, health care provider-documented diagnosis of PAD syndromes, and the ability to give informed consent. Entry criteria also included receipt of a SARS-CoV-2 vaccine within 14 days of enrollment, receipt of the second dose of mRNA vaccine (Moderna mRNA-1273 or Pfizer BioNTech BNT162b2) within 28 days of the first visit, or receipt of one dose of adenoviral-vector vaccine (J&J Ad26.COV2.S) within 35 days of an initial visit. Exclusion criteria included participation in an investigational study of SARS-CoV-2 vaccines within the past year, history of HIV infection, an active cancer diagnosis, treatment with immunosuppressive medications, history of hematologic malignancy, history of anti-CD20 monoclonal antibody therapy, receipt of live-attenuated vaccine within 30 days or any inactivated vaccine within 14 days of SARS-CoV-2 vaccination, blood or blood product donation within 30 days prior to study vaccination, and planned blood donation at any time during or 30 days after the duration of subject study participation.

In total, 469 charts were reviewed, and 160 subjects were contacted. A total of 30 adults (27 females, 3 males) with PAD syndromes met eligibility requirements and agreed to enroll in the study (see **Table S1**); we note a sex-bias in the enrollees from our PAD cohort, which is not typical for the disease itself, with 90% (n = 27) of patients in our cohort being female. Age ranged from 20 to 82, with an average age of 48.4 years old. Nineteen PAD patients had CVID with a mean IgG level of 411mg/dL (Range 177-606) and a mean protective response to Pneumovax vaccination of 5.5 serotypes (range 0-14), at the time of diagnosis. Five had hypogammaglobulinemia with a mean IgG level of 497mg/dL (Range 387-645) at the time of diagnosis. Six patients had SAD with a mean protective response to Pneumovax vaccination of 6.5 serotypes (Range 0-14), at the time of diagnosis. Twenty-seven of these subjects had received immunoglobulin replacement therapy before and during the study period from nine different products. Nineteen subjects received the BNT162b2, eight received mRNA-1273, and three received Ad26.COV2.S vaccines. Of the 30 subjects, nine were diagnosed with a prior SARS-CoV-2 infection with a positive nasal swab RT-PCR test, and one received treatment with an anti-SARS-CoV-2 monoclonal antibody (Bamlanivimab) 90 days prior to study enrollment (**Table S1**).

All subjects had one mandatory post-vaccine blood sample collection with optional pre-vaccine and follow-up visits on days 60, 90, and 150 (±14 days) after vaccination. The optional pre-vaccination blood sample was collected up to 14 days before receiving a vaccine. For subjects who received a two-dose series of mRNA vaccines, the first post-vaccination blood collection occurred 7 to 28 days after the second dose. For subjects receiving the Ad26.COV2.S single-dose vaccine, the first post-vaccination blood sample was collected 21 to 35 days after immunization. Since the study was non-interventional, patients were informed if they mounted an immune response to the vaccine, but the decision to receive a booster was made between the patient and their physician. Subjects who opted for boosting provided a blood sample up to 14 days prior to receiving the booster dose, unless the subject previously provided a sample within 2 weeks as part of the optional post-vaccine assessments. Subjects returned for an additional sample 7 to 28 days after receiving the booster (range 7-27 days, median 17 days, mean 17 days. One patient had their post-booster sample drawn on day 35). A second and third post-booster visit and sample collection occurring at 90 ± 14 days and 150 ± 14 days.

### Healthy donor cohort

Immunocompetent healthy donor volunteer blood samples were obtained as previously described (37). The healthy donor study was approved by the Institutional Review Board of Washington University School of Medicine (Approval # 202012081).

### Quantification and statistical analysis

Statistical analysis was preformed using Prism Version 9 (GraphPad). Statistical significance was determined by one-way ANOVA, unpaired t-test, mixed model analysis, or two-way ANOVA with Fisher’s least significant difference testing. Associations were calculated using Pearson rank correlation and are shown with Pearson trend lines for visualization.

Additional methods included in Supplementary materials

## Results

### Most individuals with PAD syndromes display a similar overall memory B cell response to healthy controls following primary vaccination series

**Figure 1:**
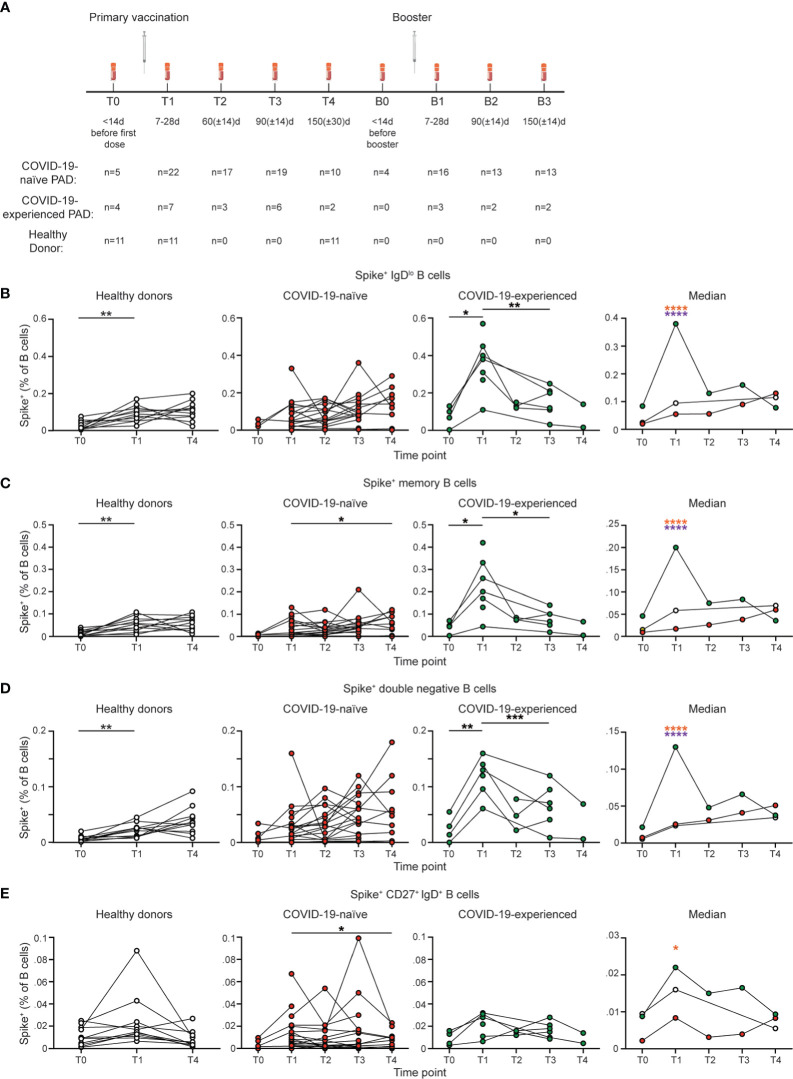
COVID-19-experienced PAD patients have elevated spike-specific B cell response following primary vaccination series. **(A)** Schematic of study design including time points in which PBMCs were obtained and number of samples per time point for each group. **(B)** Representative flow cytometry plots of the gating strategy used to identify IgD^lo^ Spike^+^ B cells. Percentage of **(C)** IgD^lo^, **(D)** memory (IgD^lo^ CD20^+^ CD38^int-lo^ CD27^+^), double negative (IgD^lo^ CD20^+^ CD38^int-lo^ CD27^-^), and **(E)** IgD^+^ CD20^+^ CD38^int-lo^ CD27^+^ Spike^+^ cells amongst the B (Live CD19^+^ CD3^-^) cell population in the healthy donor (left, white), COVID-19-naïve PAD (middle left, red), and COVID-19-experienced PAD (middle right, green) groups. Median percentage of B cells that comprise each population in all groups is shown on right. Statistical analyses in **(B–E)** were performed using a mixed-effects model (for trends found between time points) or two-way ANOVA (for trends found between groups shown on the median graphs) with Fisher’s least significant difference testing. Significance testing between time points was limited to comparisons relative to T1. Above the median graphs, an orange asterisk indicates a comparison between the COVID-19-naïve and -experienced groups, and a purple asterisk indicates a comparison between the COVID-19-experienced and healthy donor groups (*p < 0.05; **p < 0.01; ***p < 0.001; ****p < 0.001). See also **Figure S2**.

We assessed the SARS-CoV-2-specific B and CD4^+^ T cell response following vaccination in peripheral blood mononuclear cells (PBMCs) from a cohort of 30 individuals with PAD syndromes that completed their primary vaccination series (n=19 Pfizer-BioNTech BNT162b2, n=8 Moderna mRNA-1273, n=3 J&J Ad26.COV2.S) (**Figure 1A**; **Table S1**). Nine of these individuals were COVID-19-experienced with a history of acute infection and a positive SARS-CoV-2 real time-polymerase chain reaction (RT-PCR) that occurred 36 to 276 days prior to vaccination. 19 of these individuals subsequently received a booster vaccine dose (n=16 BTN162b2, n=3 mRNA-1273). PBMCs were obtained from these individuals at multiple time points following completion of the primary vaccine series or the booster immunization. PBMCs also were obtained from a separate cohort of 11 COVID-19-naïve healthy donors following completion of a primary vaccination series with Pfizer-BioNTech (BNT162b2) vaccination (**Table S1**). We used flow cytometry to assess the immune response in PBMCs following vaccination. (**Figure S1A**, **Table S2**). His-tagged spike and receptor binding domain (RBD)-binding probes identified SARS-CoV-2-specific B cells (38, 39). PAD patients had a reduced percentage of CD19^+^ B and CD3^+^ T cells relative to healthy donors (**Figures S1B, C**, **Table S2**). However, there was no difference in the percentage of IgD^lo^ B cells, memory B cells, or in the ratio of CD4^+^ to CD8^+^ cells among the T cell population (**Figures S1B, C**, **Table S2**). Overall, there was also no statistical difference in the proportion of class-switched memory cells, although some PAD patients had low levels of class-switched memory B cells (**Figure S1B**). Low levels of class-switched memory B cells are typically found in patients with more clinically severe PAD syndromes (40–42). In 28 of 30 PAD patients, the absolute lymphocyte count was within the normal range on their most recent complete blood count (**Table S3**). Six of the 26 PAD patients in our cohort with documentation of anti-Streptococcus pneumoniae titers following Pneumovax vaccination had severe phenotypes with protecting titers against 2 or fewer Streptococcus pneumoniae serotypes (**Table S4**). COVID-19-naïve patients with a poor immune response following Pneumovax vaccination (n = 5) also had reduced serum neutralizing antibody titers against WA1/2020, along with trends towards a reduced B cell response and total anti-spike antibodies following SARS-CoV-2 vaccination (**Figure S1D**).

We found that 25 of 29 (86%) PAD patients with available samples had detectable spike and RBD-specific IgD^lo^ B cell responses following vaccination (**Figures 1B**; **S2B**). Unexpectedly, most COVID-19-naïve PAD patients had SARS-CoV-2-specific B cell responses that appeared comparable to COVID-19-healthy donors at all time points tested (**Figure 1B**). The SARS-CoV-2-specific response was also comparable between PAD patients and healthy controls that received the Pfizer-BioNTech BNT162b2 vaccine (**Figure S2A**). The f4 PAD patients with no spike or RBD specific memory B cell response had reduced percentages of B cells that were IgD^lo^ and IgD^lo^ CD27^+^ and reduced SARS-CoV-2-specific total and neutralizing antibody responses following vaccination compared to responding PAD patients (**Figures S1B, E**, **Table S2**). Only 1 of the 4 PAD patients that lacked a B cell response following SARS-CoV-2 vaccination had a severe phenotype based on anti-Streptococcus pneumoniae titers. The 4 non-responding PAD patients were divided between the three vaccine groups (n=2 Pfizer-BioNTech BNT162b2, n=1 Moderna mRNA-1273, n=1 J&J Ad26.COV2.S).

COVID-19-experienced individuals with PAD syndromes had a greater SARS-CoV-2 specific B cell response at day 7 to 28 following vaccination relative to both healthy donors and COVID-19 naïve PAD patients, before declining to levels comparable to the other 2 groups by day 60 (**Figures 1B**; **S2B**). There was not a significant difference in the magnitude of the SARS-CoV-2 specific B cell response in COVID-19-naïve or experienced PAD patients when PBMCs were obtained between day 7-14, day 15-21, and day 22-28 following vaccination (**Figure S1F**). The SARS-CoV-2-specific response in the IgD^lo^ B cell (CD20^+^ CD38^int-lo^) population was divided between conventional (CD27^+^) and double negative (CD27^-^) memory B cells, with both populations displaying similar kinetics in COVID-19-naïve PAD patients and healthy donors (**Figures 1C, D**; **S2C, D**). Double negative B cells accumulate in individuals with chronic infection or autoimmunity, but also can be induced following vaccination in healthy individuals (43, 44). There was also a small population of SARS-CoV-2-specific B cells detected among IgD^+^ CD27^+^ B cells following vaccination, with this population declining to baseline levels by day 150 in all groups (**Figures 1E**; **S2E**). IgD^+^ IgM^+^ CD27^+^ B cells are sometimes referred to as circulating marginal zone B cells (45). Together, these data indicate that most individuals with PAD syndromes in our cohort had comparable B cell responses following SARS-CoV-2 vaccination to healthy donors, and that prior exposure to COVID-19 leads to a greater response upon vaccination.

### COVID-19-naïve individuals with PAD syndrome have a B cell response following booster vaccination

**Figure 2:**
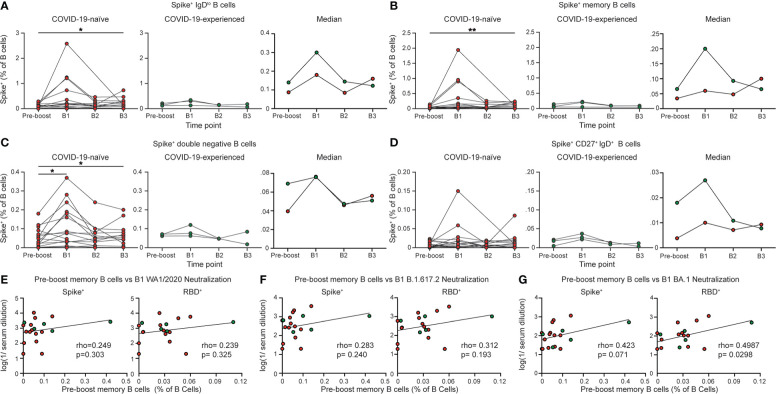
COVID-19-naïve PAD patients display elevated spike-specific B cell response following booster vaccination. Percentage of **(A)** IgD^lo^, **(B)** memory (IgD^lo^ CD20^+^ CD38^int-lo^ CD27^+^), **(C)** double negative (IgD^lo^ CD20^+^ CD38^int-lo^ CD27^-^), and **(D)** IgD^+^ CD20^+^ CD38^int-lo^ CD27^+^ Spike^+^ cells among the B (Live CD19^+^ CD3^-^) cell population in the COVID-19-naïve PAD (left, red) and COVID-19-experienced PAD (middle, green) cohorts. Median percentage of B cells that comprise each population in all groups is shown on right. Correlation between percentage of B cells that are Spike^+^ (left) or RBD^+^ (right) memory cells prior to boosting and the serum neutralizing activity against **(E)** WA1/2020, **(F)** B.1.617.2, and **(G)** BA.1. The pre-boost group consists of the last sample obtained from each patient prior to booster vaccination. Statistical analyses were performed using a mixed effects model (for trends found between time points) with Fisher’s least significant difference testing in **(A–D)**, or a Pearson rank correlation (with Pearson trend lines for visualization) in **(E–G)**. (*p < 0.05; **p < 0.01). Significance testing between time points was limited to comparisons relative to pre-boost. See also **Figure S3**.

We next evaluated the B cell response following booster vaccination in individuals with PAD syndromes. Most COVID-19-naïve individuals with PAD syndromes mounted SARS-CoV-2-specific B cell responses at day 7-28 following booster vaccination, with an elevated percentage of cells present at day 150 post-boost compared to pre-boost levels (**Figures 2A**; **S3A**). However, there were minimal increases in the SARS-CoV-2-specific B cell response in COVID-19-experienced PAD patients following boosting (**Figures 2A–D**; **S3A–D**). The SARS-CoV-2-specific B cell response following boosting was largely composed of conventional memory and double negative B cells (**Figures 2B–D**; **S3B–D**). While the percentage of SARS-CoV-2-specific memory B cells prior to booster vaccination did not correlate with the titer of neutralizing antibodies against the WA1/2020 and B.1.617.2 viruses following booster vaccination, there was a small but statistically significant correlation between the RBD-specific memory B cell response and the neutralizing antibody titer against Omicron BA.1 (**Figures 2E–G**). The spike-specific memory B cell response and the neutralizing antibody titer against BA.1 displayed a similar correlation strength but did not reach statistical significance (**Figures 2E–G**). This suggests that SARS-CoV-2-specific memory B cells present in PAD individuals prior to booster vaccination may give rise to antibody-secreting cells capable of neutralizing some viral variants.

### IgG1 class-switching defect in memory B cells from COVID-19-naïve individuals with PAD syndrome is rescued following booster vaccination

**Figure 3:**
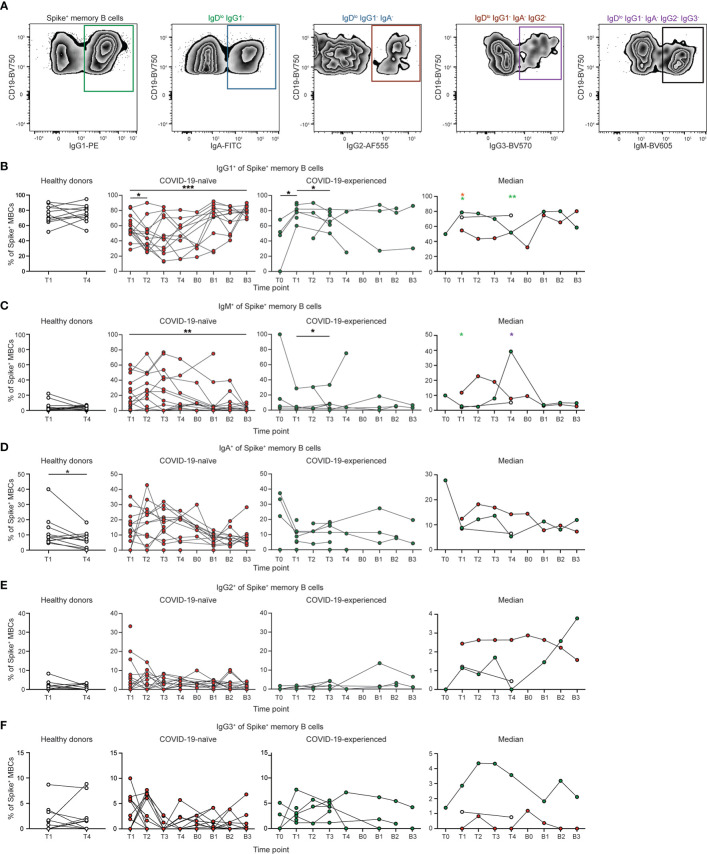
Spike-specific memory B cells from COVID-19-naïve PAD patients display reduced IgG1 class switching following primary vaccination series. **(A)** Representative flow cytometry plots of the gating strategy used to identify the immunoglobulin subclass of Spike^+^ memory (IgD^lo^ CD20^+^ CD38^int-lo^ CD27^+^) B cells. Percentage of Spike^+^ memory B cells that are **(B)** IgG1^+^, **(C)** IgM^+^, **(D)** IgA^+^, **(E)** IgG2^+^, and **(F)** IgG3^+^ in healthy donors (left, white), and COVID-19-naïve (middle left, red) and -experienced (middle right, green) PAD cohorts. Median percentage of B cells that comprise each population in all groups is shown on right. Statistical analyses in B-D were performed using a mixed effects model (for trends found between time points) or two-way ANOVA (for trends found between groups shown on the median graphs) with Fisher’s least significant difference testing. Significance testing between time points was limited to comparisons relative to T1. Above the median graphs, a green asterisk indicates a comparison between the COVID-19-naïve and healthy donor groups, an orange asterisk indicates a comparison between the COVID-19-naïve and -experienced groups, and a purple asterisk indicates a comparison between the COVID-19-experienced and healthy donor groups (*p < 0.05; **p < 0.01; ***p < 0.001). See also **Figure S4**.

We next evaluated the immunoglobulin subclass specificity of the conventional memory B cell response following SARS-CoV-2 vaccination in the 25 PAD patients that responded to vaccination (**Figure 3A**). IgD^lo^ B cells that have positive staining by flow cytometry for an immunoglobulin subclass displayed minimal staining for other subclasses (**Figure S4A**). COVID-19-naïve individuals with PAD syndromes had reduced percentages of IgG1^+^ memory B cells relative to healthy donors at day 7 to 28 post-vaccination, with these individuals also displaying an elevated percentage of IgM^+^ SARS-CoV-2-specific memory B cells (**Figures 3B, C**; **S4B, C**). However, booster vaccination led to an increase in the percentage of IgG1^+^ and decrease in the percentage of IgM^+^ spike-specific memory B cells in COVID-19-naïve PAD patients to levels comparable to that seen in healthy donors post primary vaccination (**Figures 3B, C**; **S4B, C**). There was no significant difference in the percentage of IgA^+^, IgG2^+^ or IgG3^+^ SARS-CoV-2-specific memory B cells between healthy donors and COVID-19-naïve individuals (**Figures 3D, F**; **S4D–F**). COVID-19-experienced individuals with PAD syndromes displayed a similar memory B cell immunoglobulin subclass composition to the healthy donor cohort following the primary vaccination series (**Figures 3**; **S4**). Together, these data indicate that the repeated exposure to SARS-CoV-2 through vaccination and/or infection can rescue defects in IgG1-class switching seen in some individuals with PAD syndrome.

### Memory B cells from individuals with PAD syndrome display impaired CD11c expression following vaccination

**Figure 4:**
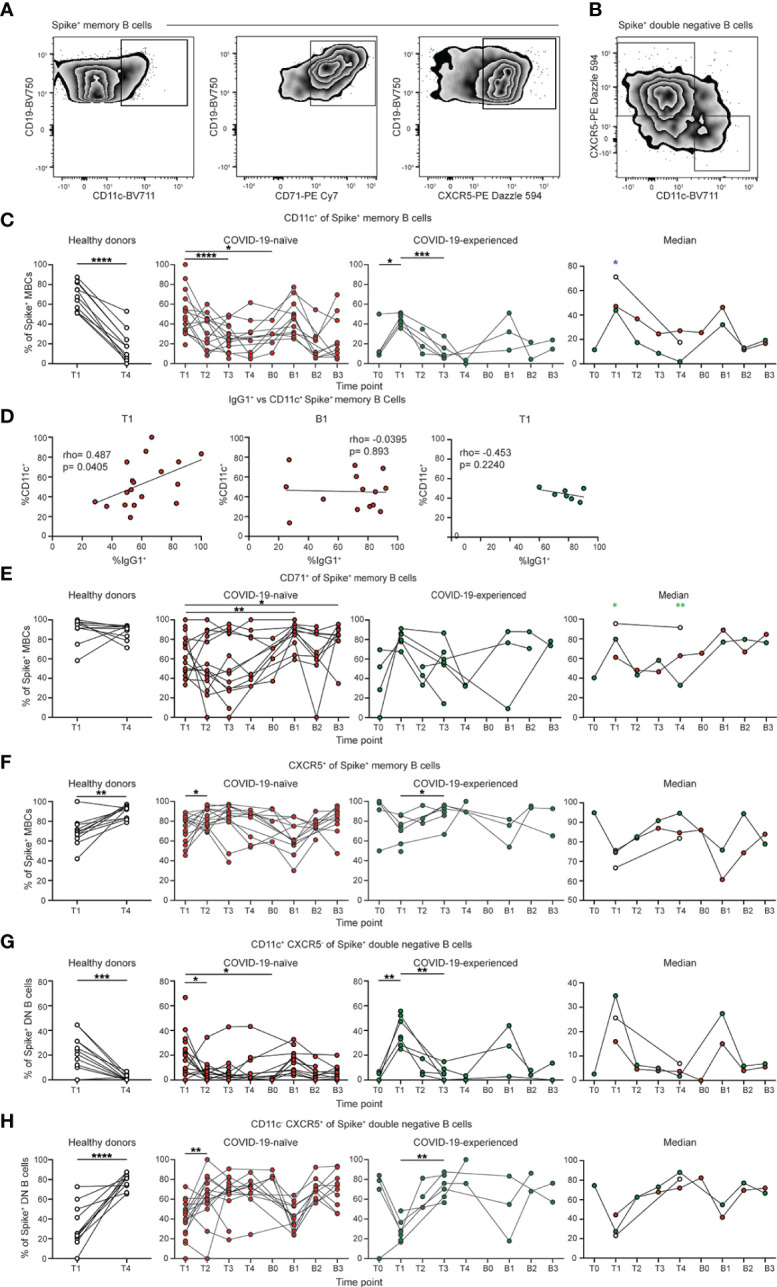
Spike-specific memory B cells from PAD patients display reduced CD11c expression. **(A)** Representative flow cytometry plots of the expression of CD11c, CD71, and CXCR5 on Spike^+^ memory (IgD^lo^ CD20^+^ CD38^int-lo^ CD27^+^) B cells. **(B)** Representative flow cytometry plots of the expression of CD11c and CXCR5 on Spike^+^ double negative (IgD^lo^ CD20^+^ CD38^int-lo^ CD27^-^) B cells. **(C)** Percentage of Spike^+^ memory B cells that are CD11c^+^ in the healthy donor (left, white), COVID-19-naïve PAD (middle left, red), and COVID-19-experienced PAD (middle right, green) cohorts. Median percentage of CD11c^+^ cells in all groups is shown on right. **(D)** Correlation between percentage of Spike^+^ memory B cells that are IgG1^+^ and CD11c^+^ at T1 (left) and B1 (middle) in the COVID-19-naïve PAD patients, and at T1 (right) in the. COVID-19-experienced PAD patients. Associations for **D** are calculated using Pearson rank correlation and shown with Pearson trend lines for visualization. Percentage of Spike^+^ memory B cells that are **(E)** CD71^+^ or **(F)** CXCR5^+^ in healthy donors (left, white), COVID-19-naïve PAD (middle left, red), and COVID-19-experienced PAD (middle right, green) cohorts. Median percentage of B cells that comprise each population in all groups is shown on right. Percentage of Spike^+^ double negative B cells that are **(G)** CD11c^+^ CXCR5^-^ or **(H)** CD11c^-^ CXCR5^+^ in the healthy donor (left, white), COVID-19-naïve PAD (middle left, red), and COVID-19-experienced PAD (middle right, green) cohorts. Median percentage of double negative B cells that comprise each population in all groups is shown on right. Statistical analyses in **(C, E–H)** were performed using a mixed effects model (for trends found between time points) or two-way ANOVA (for trends found between groups shown on the median graphs) with Fisher’s least significant difference testing. Significance testing between time points was limited to comparisons relative to T1. Above the median graphs, a green asterisk indicates a comparison between the COVID-19-naïve and healthy donor groups, an orange asterisk indicates a comparison between the COVID-19-naïve and COVID-19-experienced groups, and a purple asterisk indicates a comparison between the COVID-19-experienced and healthy donor groups (*p < 0.05; **p < 0.01; ***p < 0.001; ****p < 0.0001). See also **Figure S5**.

We next assessed the phenotype of the SARS-CoV-2 specific conventional memory B cell response post vaccination (**Figures 4A**; **B** **)**. We observed high levels of expression of CD11c on SARS-CoV-2-specific memory B cells at day 7 to 28 following vaccination in all groups (**Figures 4C**; **S5A**). CD11c expression is induced on B cells following antigen encounter (46). There was also an increase in the percentage of CD11c^+^ cells following boosting in the COVID-19-naïve group (**Figures S4C, S5A**). However, CD11c expression in COVID-19-naïve and experienced PAD patients was reduced relative to healthy donors at day 7 to 28 post primary vaccination (**Figures 4C**; **S5A**). Boosting did not lead to an increase in the expression of CD11c in PAD patients compared to the day 7 to 28 time point. There was a correlation between the percentage of CD11c^+^ and IgG1^+^ Spike^+^ memory B cells at day 7 to 28 post vaccination in COVID-19-naïve PAD patients (**Figures 4D**; **S5B**). B cell receptor (BCR) signaling can cooperate with CD40 and toll-like receptor signaling pathways to induce IgG1 class-switching (47, 48). Thus, the defect in BCR signaling indicated by reduced CD11c expression could result in an impaired ability of other signaling pathways to induce IgG1 class switching in some PAD patients following vaccination (47, 48). The lack of correlation between the percentage of CD11c^+^ and IgG1^+^ CD27^+^ Spike^+^ memory B cells following boosting or in the COVID-19-experienced PAD patients suggests that the defect in BCR signaling and IgG1 class-switching seen following primary vaccination might be overcome by additional costimulatory signals (**Figures 4D**; **S5B**). The expression of CD71, a marker of activated B cells, was also reduced in COVID-19-naïve PAD patients relative to healthy donors post vaccination (49) (**Figures 4E**; **S5C**). The percentage of CD11c^+^ memory B cells decreased by day 90 post vaccination or boosting, and this was accompanied by a concomitant increase in the percentage of CXCR5^+^ memory B cells in al groups (**Figures 4C, F**; **S5A, D**). Together, these data suggest that some PAD patients may exhibit impaired BCR signaling following vaccination.

We also determined the phenotype of the SARS-CoV-2-specific double negative B cell response following vaccination. Double negative B cells can be divided into subsets based on the expression of CD11c and CXCR5 (50). There was an increased percentage of CD11c^+^ CXCR5^-^ (DN2) cells at day 7 to 28 post vaccination in all groups, with a higher level in COVID-19 experienced than COVID-19-naïve PAD patients (**Figures 4G**; **S5E**). A similar increase was observed in both PAD cohorts following boosting (**Figures 4G**; **S5E**). Conversely, there was a decreased percentage of CD11c^-^ CXCR5^+^ (DN1) cells at day 7 to 28 post vaccination in all groups, with this percentage increasing at day 60 post vaccination (**Figures 4H**; **S5F**). A similar decrease in the percentage of DN1 cells also was apparent following boosting in PAD patients (**Figures 4G**; **S5E**). There was no clear difference in the phenotype of the SARS-CoV-2-specific double negative memory B cells between the COVID-19-naïve PAD individuals and the healthy donor group (**Figures 4G, H**; **S5E, F**).

### Booster vaccination induces Omicron-specific memory B cells in individuals with PAD syndrome

**Figure 5:**
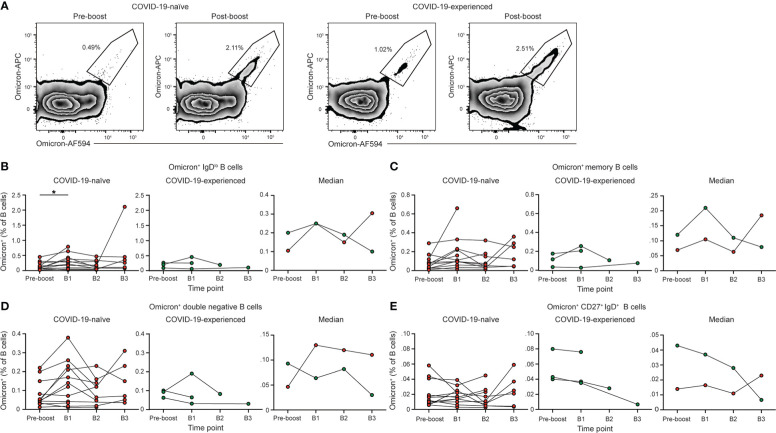
PAD patients have elevated Omicron-specific B cell responses following booster vaccination. **(A)** Representative flow cytometry plots of the percentage of Omicron-specific B cells among the memory (IgD^lo^ CD20^+^ CD38^int-lo^ CD27^+^) B cell population prior to and post booster vaccination in COVID-19-naïve (left) and -experienced (right) individuals with PAD syndromes. Percentage of **(B)** IgD^lo^, **(C)** memory, **(D)** double negative (IgD^lo^ CD20^+^ CD38^int-lo^ CD27^-^), and **(E)** IgD^+^ CD20^+^ CD38^int-lo^ CD27^+^ Omicron^+^ cells among the B (Live CD19^+^ CD3^-^) cell population in healthy donors (left, white), COVID-19-naïve PAD (middle left, red), and COVID-19-experienced PAD (middle right, green) cohorts. Median percentage of B cells that comprise each population in all groups is shown on right. Statistical analyses in **(B–E)** were performed using a mixed effects model (for trends found between time points) or two-way ANOVA (for trends found between groups shown on the median graphs) with Fisher’s least significant difference testing. Significance testing between time points was limited to comparisons relative to pre-boost. Above the median graphs, an orange asterisk indicates a comparison between the COVID-19-naïve and -experienced groups (*p < 0.05). See also **Figure S6**.

We assessed the Omicron-specific B cell response in individuals with PAD syndromes that responded to primary vaccination using a His-tagged protein specific for the spike protein of BA.1 (**Figure 5A**). Administration of a homologous booster vaccine led to an increase in the percentage of Omicron-specific memory B cells in COVID-19-naïve individuals (**Figure 5B**). The percentage of Spike^+^ memory B cells post booster vaccination was similar when stained with probes targeting the ancestral and Omicron spike protein (**Figure S6**). This increase was evident in both the conventional and double negative B cell populations (**Figures 5C, D**), although there was no increase in Omicron-specific IgD^+^ CD27^+^ B cells (**Figure 5E**). The percentage of Omicron-specific B cells returned to pre-boost baseline level in most COVID-19-naïve individuals by day 90 post-boost (**Figures 5B–D**). However, patients 102 and 110 displayed an increase in their percentage of Omicron-specific B cells between 90 and 150 days post-booster vaccination. This time point coincided with the period between November 2021 and January 2022, when COVID-19 cases in the United States surged due to the emergence of the Omicron variant. Patient 102 was confirmed as having been re-infected between B2 and B3. Of note, patients 102 and 110 reported no or very mild symptoms during this period.

### Individuals with PAD syndrome have SARS-CoV-2-specific CD4^+^ T cell responses following vaccination

**Figure 6:**
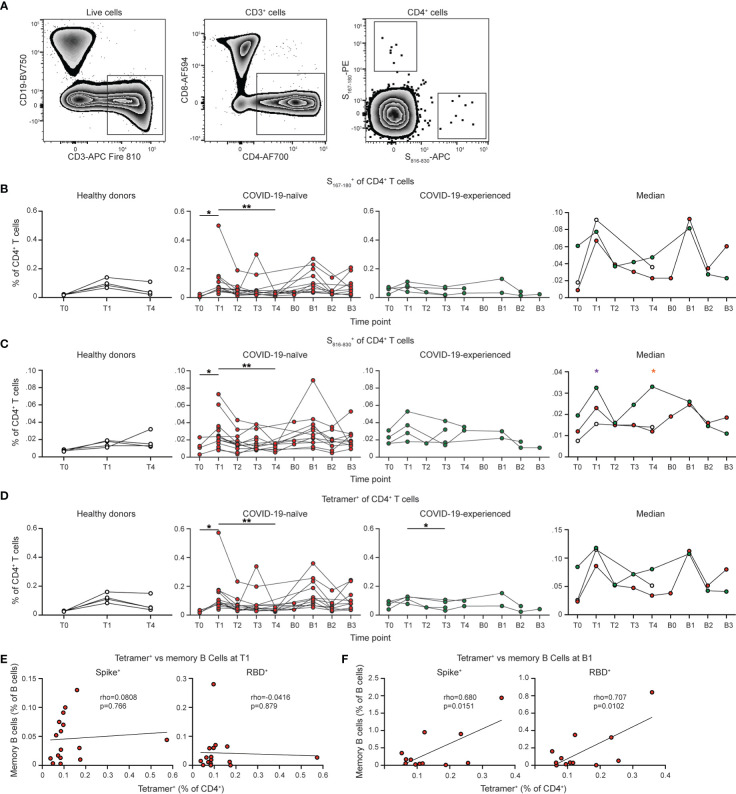
PAD patients display unimpaired SARS-CoV-2-specific CD4^+^ T cell responses following vaccination. **(A)** Representative flow cytometry plots of the gating strategy used to identify S_167-180_^+^ and S_816-830_^+^ CD4^+^ T cells. Percentage of CD4^+^ (Live CD3^+^ CD19^-^ CD4^+^ CD8^-^) T cells that are **(B)** S_167-180_^+^, **(C)** S_816-830_^+^, or **(D)** Tetramer^+^ (S_167-180_^+^ or S_816-830_^+^) in healthy donors (left, white), COVID-19-naïve PAD (middle left, red), and COVID-19-experienced PAD (middle right, green) cohorts. Median percentage of CD4^+^ T cells that comprise each population in all groups is shown on right. Correlation between percentage of B cells that are Spike^+^ (left) or RBD^+^ (right) memory (IgD^lo^ CD20^+^ CD38^int-lo^ CD27^+^) cells and the percentage of CD4^+^ T cells that are Tetramer^+^ at **(E)** T1 or **(F)** B1. Associations for **(E, F)** are calculated using Pearson rank correlation and shown with Pearson trend lines for visualization. Statistical analyses in **(B–D)** were performed using a mixed effects model (for trends found between time points) or two-way ANOVA (for trends found between groups shown on the median graphs) with Fisher’s least significant difference testing. Significance testing between time points was limited to comparisons relative to T1. Above the median graphs, an orange asterisk indicates a comparison between the COVID-19-naïve and COVID-19-experienced groups, and a purple asterisk indicates a comparison between the COVID-19-experienced and healthy donor groups (*p < 0.05; **p < 0.01). .

We also evaluated the CD4^+^ T cell responses following SARS-CoV-2 vaccination (**Figure 6A**). SARS-CoV-2-specific CD4^+^ T cells were identified using a S_167-180_ tetramer, which binds an immunodominant SARS-CoV-2 spike epitope restricted by the HLA-DPB1*04:01 allele that is found at >40% frequency in many populations (51). We also developed a S_816-830_ tetramer that is specific for an immunodominant region of the S2 portion of the spike protein (52, 53). This region is highly conserved among coronaviruses and also restricted to the HLA-DPB1*4:01 allele (52). 16 of 30 individuals with PAD syndromes and 4 of 11 healthy donor samples had detectable SARS-CoV-2-specific CD4^+^ T cell responses. COVID-naïve PAD patients had a similar percentage of SARS-CoV-2-specific CD4^+^ T cell response as healthy donors at day 7 to 28 post vaccination with this response contracting by day 150 in both groups (**Figures 6B–D**). Boosting led to an increase in the SARS-CoV-2-specific CD4^+^ T cell response (**Figures S6B–D**). COVID-experienced PAD patients had increased percentages of SARS-CoV-2-specific CD4^+^ T cell prior to vaccination relative to the other groups, consistent with a pre-existing memory response (**Figures 6B–D**). There was no correlation between the magnitude of the SARS-CoV-2 specific CD4^+^ T cell response and the conventional memory B cell response following vaccination in PAD patients (**Figure 6E**). However, there was a small but statistically significant correlation following boosting suggesting that the pre-existing memory response in PAD patients can give rise to enhanced B and T cell responses following antigen re-encounter (**Figure 6F**).

### SARS-CoV-2-specific CD4^+^ T cells from PAD patients are phenotypically similar to cells from healthy donors

**Figure 7:**
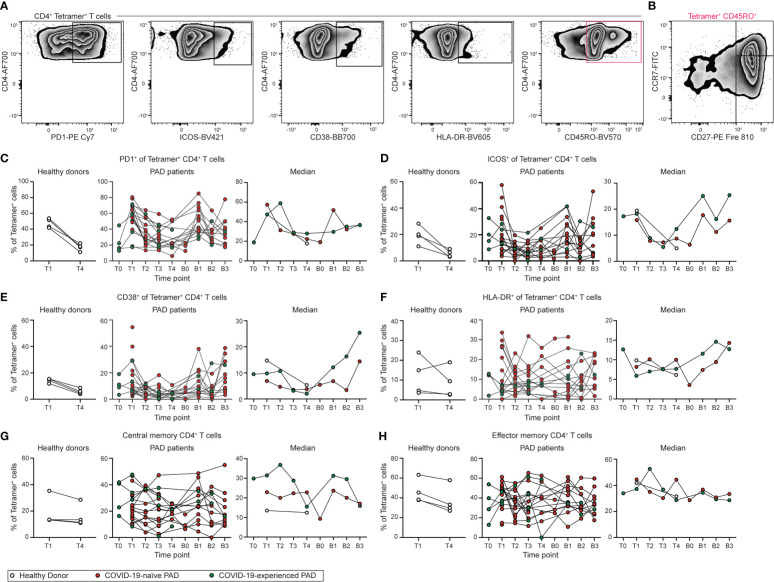
Phenotype of SARS-CoV-2-specific CD4^+^ T cell response following vaccination in PAD patients. **(A)** Representative flow cytometry plots of the expression of PD1, ICOS, CD38, HLA-DR, and CD45RO on Tetramer^+^ CD4^+^ (Live CD3^+^ CD19^-^ CD4^+^ CD8^-^ S_167-180_^+^ or S_816-830_^+^) T cells. **(B)** Representative flow cytometry plots of the expression of CD27 and CCR7 on CD45RO^+^ Tetramer^+^ CD4^+^ T cells. Percentage of Tetramer^+^ CD4^+^ T cells that are **(C)** PD1^+^, **(D)** ICOS^+^, **(E)** CD38^+^, **(F)** HLA-DR+, **(G)** central memory (CD45RO^+^ CD27^+^ CCR7^+^), or **(H)** effector memory (CD45RO^+^ CD27^+^ CCR7^-^) in healthy donors (left, white), COVID-19-naïve PAD (middle, red), and COVID-19-experienced PAD (middle, green) cohorts. Median percentage of Tetramer^+^ T cells that that comprise each population in all groups is shown on right. See also **Figure S7**.

The phenotype of the SARS-CoV-2-specific CD4^+^ T cell response was next assessed (**Figures 7A, B**; **S7A**). SARS-CoV-2-specific CD4^+^ T cells adopted an activated phenotype following vaccination and boosting in PAD patients, with this response characterized by increased cell surface expression of PD1, ICOS, and CD38 (**Figures 7C–E**; **Figures S7B, C**). Expression of PD1, ICOS, and CD38 were similar between COVID-19-naïve individuals with PAD syndromes and healthy donors at day 7 to 28 following vaccination, suggesting there was no defect in CD4^+^ T cell activation in most individuals with PAD syndromes (**Figures 7C–E**). We also did not detect a difference in HLA-DR expression between any of the groups (**Figures 7F**; **S7C**). Circulating T_FH_ (CXCR5^+^PD1^+^) cells were not identified in most samples, consistent with previous work demonstrating that these cells are detectable in the blood only one week post vaccination (**Figure S7D**) (51). The phenotype of the CD4^+^ T cell response was assessed further by determining the percentage of SARS-CoV-2-specific central (CD45RO^+^CD27^+^CCR7^+^) and effector (CD45RO^+^CD27^+^CCR7^+^) memory CD4^+^ T cells (**Figures 7G, H**). No difference was observed in the composition of the memory T cell response at T4 between the groups, suggesting the development of memory CD4^+^ T cells in PAD patients is intact (**Figures 7G, H**). Together, these data indicate that patients with PAD syndromes in our cohort that has a detectable response developed SARS-CoV-2-specific CD4^+^ T cells that were similar in magnitude and phenotype to those measured in healthy donors following vaccination.

## Discussion

In this study, we show that SARS-CoV-2 vaccination induces a long-lived memory B and CD4^+^ T cell response in most individuals with PAD syndromes that is comparable to the response seen in healthy donors. Only 4 of 29 PAD patients failed to develop a spike-specific memory B cell response following vaccination, and these individuals also had lower levels of bulk memory and IgD^lo^ B cells compared to the other patients in the cohort. These results suggest that memory B and T cells can promote long-term protective immunity in many individuals with PAD syndromes, despite their history of recurrent infections, hypogammaglobulinemia, and impaired ability to mount optimal and sustained antibody responses after immunization with other vaccines.

The lack of defect in the memory B cell response contrasts with our previous findings that COVID-19 naïve PAD patients display an impaired SARS-CoV-2-specific antibody response following vaccination (26). Differences between the magnitude of the memory B cell and serum antibody response in PAD patients might reflect the distinct signals needed for induction and survival of memory B cells compared to antibody-secreting cells. For example, short-lived plasmablasts develop from a germinal-center independent extrafollicular response, whereas long-lived memory B cells are predominantly derived from the germinal center (54). Plasmablast differentiation is impaired in some CVID patients after T-dependent and independent vaccination (55).

Despite the overall equivalent memory B cell response in PAD patients in our cohort, SARS-CoV-2-specific memory B cells from COVID-19-naïve PAD patients displayed reduced IgG1 class-switching following the primary vaccination series. IgG1 and IgG3 are the dominant isotypes elicited after viral infection with spike-specific IgG1 most closely correlated with *in vitro* SARS-COV-2 neutralization (56). This suggests that in the absence of boosting, memory B cells from some PAD patients may have an impaired ability to give rise to protective antibodies upon re-infection. This defect is further compounded by the overall decrease in SARS-CoV-2-specific neutralizing antibodies in these individuals (26). Memory B cells from PAD patients also displayed impaired CD11c expression, with a positive correlation seen between the percentage of spike-specific CD11c^+^ and IgG1^+^ cells following vaccination. CD11c expression is regulated by BCR stimulation, with CD11c^+^ B cells displaying an increased expression of genes involved in B cell activation and antigen presentation (46). Other signals including toll-like receptors and cytokines can also drive the accumulation of CD11c^+^ B cells (57–59). Although further studies are warranted, this suggests that the defect in IgG1 class-switching in some PAD patients may be due to impaired BCR signaling and/or reduced T cell help (59).

IgG1 class switching was rescued in COVID-19-naïve PAD patients following booster vaccination, which also induced an increase in Omicron-specific B cells. These data indicate that administration of mRNA booster vaccination doses may have durable benefits in addition to the short-term increase in total SARS-CoV-2-specific B cells, serum antibody titers, and neutralizing activity (26). However, COVID-19 experienced individuals with PAD syndrome displayed no further increase in SARS-CoV-2-specific B cells after boosting. These individuals also did not display any further increase in their percentage of IgG1^+^ memory B cells or in antibody avidity (26). This finding is consistent with other studies showing that COVID-19-experienced healthy donors do not increase their SARS-CoV-2-specific memory B cell response following boosting (60). However, booster vaccination may still promote additional evolution of the memory B cell repertoire in COVID-19-experienced individuals with PAD syndromes that is independent of cell number or the antibody response (61).

When the B cell response after completion of the primary vaccination series of PAD patients was assessed in other studies, they did not detect a SARS-CoV-2-specfic memory B cell response above baseline and concluded that vaccination of individuals with PAD syndromes primarily results in a double negative memory B cell response (62, 63). We find that SARS-CoV-2 vaccination induces both a conventional and double negative memory B cell response in most PAD patients that is comparable to healthy donors, and that this response is maintained for at least 150 days after completion of the primary vaccination series. The disparity in results between studies may be due to a difference in sensitivity of the probes used, as the previous study detected very few cells that bound to spike probes even in healthy donors. Heterogeneity existing within individuals classified as having PAD syndromes also may contribute to differences seen between these studies as cohorts with higher percentages of individuals with more severe phenotypes may exhibit more pronounced B cell defects following SARS-CoV-2 mRNA vaccination (64). However, the similarity in the SARS-CoV-2-specific antibody defect found between our cohort and others, and the normal memory B cell response found in some patients with a severe phenotype suggest that many individuals with PAD syndromes will benefit from SARS-CoV-2 vaccination (17, 25, 26).

Most individuals with PAD syndromes receive immunoglobulin replacement therapy. Immunoglobulin replacement products administered between May 2021- January 2022 had low levels of SARS-CoV-2-specific antibody titers with low neutralization activity (26). While the titer of anti-spike and anti-RBD in immunoglobulin replacement products has increased over time, the long lag of 9-12 months between collection, production, and distribution may make most available immunoglobulin replacement products less effective against current circulating Omicron variants (36). Although many individuals with PAD might be eligible for long-acting combination monoclonal antibody prophylaxis (e.g., Evusheld [AZD7442]) against COVID-19, recent studies showed substantial losses in potency against many lineages of the Omicron virus (BA.1, BA.2.12.1, B.A.2.75, BA.4, BA.5) (65–67). Therefore, immunization of individuals with PAD syndromes with mRNA vaccines that include a booster may be an effective way to induce a protective immune response against SARS-CoV-2 and its variants.

T cells also have an important role in mediating protective immunity against SARS-CoV-2 (54). Assessment of the SARS-CoV-2-specific T cell response typically involves stimulation of PBMCs with peptides spanning the SARS-CoV-2 spike protein and assaying for activation induced markers (AIM) or intracellular cytokines. Previous studies reported a reduced percentage of interferon gamma-producing T cells following *ex vivo* stimulation in some vaccinated individuals with PAD syndromes (17, 25, 63). We stained PBMCs with tetramers against both S_167-180_ and S_816-830_ tetramers, which bind to immunodominant peptides restricted by the HLA-DPB1*04:01 allele. This allowed us to directly detect SARS-CoV-2 specific CD4^+^ T cells without requiring additional stimulation. The SARS-CoV-2-specific CD4^+^ T cell response was comparable in magnitude between COVID-naïve PAD patients and healthy donors that generated a detectable response. Booster vaccination led to an increase in the SARS-CoV-2-specific CD4^+^ T cell response. While the CD4^+^ T cell phenotypes were comparable between groups, this does not exclude the possibility that SARS-CoV-2-specific CD4+ T cells in some individuals with PAD syndromes could exhibit impaired cytokine production. Further work is needed to assess the magnitude and functional phenotype of the SARS-CoV-2-specific CD8^+^ T cell response in PAD patients.

### Limitations to the study

Not all patients elected to receive a booster vaccination resulting in a reduced number of samples in the post-booster time points. This is particularly apparent in the COVID-experienced group, which included only three individuals that received booster vaccinations. The design of this study precluded the collection of a pre-vaccination blood draw from all individuals limiting the statistical power of comparisons made to the T0 time point. Logistical issues regarding when patients were available for blood draws also limited the precision of the time point in which the post vaccination samples were obtained. Additionally, post booster samples were not available for the healthy donor cohort, with this cohort also displaying differences in age and sex distribution relative to the PAD cohort. The low number of antigen-specific B and T cells in some samples may also contribute to variability in the assessed phenotypes. Finally, it is important to note that PAD syndromes are a heterogeneous group of diseases with our cohort including individuals with CVID, hypogammaglobulinemia, and specific antibody deficiency. While we did not observe a clear difference in the immune response between these subgroups, there could be heterogeneity between different PAD subgroups depending on the severity of disease that necessitates different vaccination and boosting approaches.

## Data availability statement

The raw data supporting the conclusions of this article will be made available by the authors, without undue reservation.

## Ethics statement

The studies involving human participants were reviewed and approved by Institutional Review Board of Washington University School of Medicine (Approval # 202104138). The patients/participants provided their written informed consent to participate in this study.

## Author contributions

FL performed experiments, analyzed the data, and prepared the manuscript. AD enrolled subjects, collected demographic and clinical data, processed PBMC samples, performed experiments, and analyzed data. HD-A, FZ, and ER processed PBMC samples. LA and DF generated crucial reagents. CH performed experiments and analyzed the data. C-YL, LV, and RC designed and performed antibody neutralization experiments and analyzed data. JM and CR collected demographic and clinical data, enrolled patients, and provided patient care. HW, AKau, TD, AKul and ZR provided patient care. TM wrote the study protocol, managed IRB compliance, enrolled patients, and processed patient samples. CO’S collected patient demographic and clinical data, enrolled patients, and processed samples. SR planned experiments and analyzed data. AE and JT contributed samples from the healthy donor cohort. JO’H and RP wrote and maintained the Institutional Review Board protocol, recruited participants, phlebotomized participants, and coordinated sample collection of healthy donors. PK supervised the project. PM generated crucial reagents. MD planned experiments, prepared the manuscript, and analyzed data. OZ designed the study, wrote the study protocol, processed PBMC samples, performed experiments, analyzed the data, prepared the manuscript, and supervised the project. BL planned the experiments, analyzed the data, prepared the manuscript, and supervised the project. All authors contributed to the article and approved the submitted version.
