# Supplementary material for: SARS-CoV-2 booster vaccination rescues attenuated IgG1 memory B cell response in primary antibody deficiency patients
Source: Front Immunol. 2022 Dec 22;13:1033770. doi: 10.3389/fimmu.2022.1033770 (PMC9817149; doi:10.3389/fimmu.2022.1033770)
Supplement: Supplementary file 1 [file DataSheet_1.pdf]

Supplementary Material for

SARS-CoV-2 booster vaccination rescues attenuated IgG1 memory B cell response in primary antibody deficiency patients

Frank J. Lin[1,9], Alexa Michelle Altman Doss[2,9], Hannah G. Davis-Adams[1], Lucas J. Adams[3], Christopher H. Hanson[1], Laura A. VanBlargan[1], Chieh-Yu Liang[1,3], Rita. E. Chen[1,3], Jennifer Marie Monroy[1], H. James Wedner[1], Anthony Kulczycki[1], Tarisa L. Mantia[1], Caitlin C. O'Shaughnessy[1], Saravanan Raju[3], Fang R. Zhao[1], Elise Rizzi[1], Christopher J. Rigell[1], Tiffany Biason Dy[1], Andrew L. Kau[1,4], Zhen Ren[1], Jackson S. Turner[3], Jane A. O'Halloran[1], Rachel M. Presti[1, 6], Daved H. Fremont[3], Peggy L. Kendall[1], Ali H. Ellebedy[3, 5, 6], Philip A. Mudd[6, 8], Michael S. Diamond[1,3,5,6, 7], Ofer Zimmerman*[1], and Brian J. Laidlaw*[1]

Affiliations:
[1] Department of Medicine, Washington University School of Medicine, St. Louis, MO, USA.
[2] Department of Pediatrics, Washington University School of Medicine, St. Louis, MO, USA.
[3] Department of Pathology and Immunology, Washington University School of Medicine, St. Louis, MO, USA.
[4] Center for Women's Infectious Disease Research, Washington University School of Medicine, St. Louis, MO, USA.
[5] The Andrew M. and Jane M. Bursky Center for Human Immunology & Immunotherapy Programs, Washington University School of Medicine, Saint Louis, MO, USA.
[6] Center for Vaccines and Immunity to Microbial Pathogens, Washington University School of Medicine, Saint Louis, MO, USA.
[7] Department of Molecular Microbiology, Washington University School of Medicine, St. Louis, MO, USA.
[8] Department of Emergency Medicine, Washington University School of Medicine, St. Louis, MO, USA.
[9] These authors contributed equally to this work and share first authorship

* Correspondence should be addressed to Brian Laidlaw (brian.laidlaw@wustl.edu) and Ofer Zimmerman (zimmero@wustl.edu)

This PDF includes:

Supplementary methods
Supplementary references
Fig. S1. Baseline phenotype of PAD patients and healthy donor cohorts
Fig S2. COVID-19-experienced PAD patients have an elevated RBD-specific B cell response following primary vaccination series
Fig. S3. COVID-19-naïve PAD patients have an elevated RBD-specific B cell response following booster vaccination
Fig. S4. Isotype composition of SARS-CoV-2 RBD-specific memory B cell response following vaccination in PAD patients
Fig. S5. RBD-specific memory B cells from PAD patients display reduced CD11c expression class.
Fig. S6. Booster vaccination induces similar percentages of Ancestral and Omicron Spike$^+$ B cells
Fig. S7. Additional phenotyping of SARS-CoV-2-specific CD4$^+$ T cell response following vaccination in PAD patients

Table S1. Characteristics of patient cohort
Table S2. Cellular phenotype of patient cohort
Table S3. PAD patient laboratory values
Table S4. PAD patient responses to other vaccine antigens at time of diagnosis

SUPPLEMENTARY METHODS

Diagnosis criteria for primary antibody deficiency syndromes cohort. Participant records were reviewed to verify that they met criteria for the diagnosis of CVID, hypogammaglobulinemia or SAD. CVID was defined by history of recurrent infections and other clinical features of CVID (e.g., autoimmunity, pulmonary or GI disease), low IgG (normal range 700-1600 mg/dL), with low IgA (normal range 70-400 mg/dL) or low IgM (normal range 40-230 mg/dL) levels and poor or absent response to vaccination, without apparent secondary causes noted at diagnosis or during early follow-up (68,69). Hypogammaglobulinemia was defined by recurrent infections with low IgG and normal response to vaccination. SAD was defined by recurrent infections and inadequate antibody response to polysaccharide antigens with normal response to protein antigens and normal serum levels of immunoglobulins (IgG, IgA, and IgM). Due to the retrospective nature of this work, we accepted the diagnosis of CVID in patients that had typical clinical features and were responsive to immunoglobulin replacement therapy even if IgA or IgM levels were both normal, as long as IgG levels were significantly reduced (IgG < 550 mg/dL) and the response to Pneumovax vaccination was poor (70). We also accepted the clinical diagnosis in one patient that was diagnosed with CVID decades ago with undetectable levels of IgA and IgM where we could not obtain pre-intravenous immunoglobulin (IVIG) therapy IgG levels or anti-*Streptococcus pnuemoniae* titers. A diagnosis of CVID was also accepted in patients with extremely low levels of IgG (n=4; Range 177-346 mg/dL) and low levels of IgA and IgM who were started on IVIG without an assessment of their response to vaccination.

Preparation of peripheral blood mononuclear cells. Patient blood was collected in sodium citrate cell preparation tubes (BD Biosciences). Tubes were centrifuged at room temperature for 30 min at 1650 x *g* without brake. The mononuclear cells and plasma layer were then transferred to a 50 mL conical tube and centrifuged at room temperature for 10 min at 256 x *g*. Supernatant was removed without disturbing the cell pellet. Cells were resuspended in PBS, and all cells of the same subject were combined into one 15-mL conical tube. Cells were centrifuged as above. Supernatant was removed without disturbing the cell pellet, and cells were resuspended in 5 mL of ACK lysing buffer (Thermo Fisher) for 5 min. Cells were washed with PBS, counted, washed again with PBS, resuspended in 10% dimethylsulfoxide in FBS at 10^6 cells per mL and aliquoted into cryovials. Cryovials were transferred to a freezing container (Daigger Scientific, Mr. Frosty) and placed in a -80°C freezer overnight before being transferred to liquid nitrogen for storage.

Antibodies for flow cytometry staining. Staining for flow cytometry was performed using cryopreserved PBMC samples. Cryopreserved samples were thawed in a 37°C water bath and added to tubes containing RPMI with 10% FBS and 1% penicillin/streptomycin solution (Fisher). Cells then were centrifuged at 300 x *g* at room temperature, resuspended in 10% RPMI, and counted. 3 x 10^6 cells were then aliquoted to a 96 well plate and centrifuged at 810 x *g* for 2 min at 4°C. The cells were then washed with PBS containing 2% FBS, centrifuged, and then incubated with the primary antibody mix for 30 min on ice. Samples that were stained with a tetramer-containing antibody cocktail were incubated for 45 min. Cells were then washed three times with 2% PBS and incubated with secondary antibody mix for an additional 30 min. Samples then were washed an additional three times with 2% PBS before being resuspended for flow cytometry analysis. Flow cytometry data were acquired on a Cytek Aurora and were analyzed with FlowJo software (TreeStar).

The following antibodies were used for flow cytometry staining: Brilliant Violet 711 (BV711) anti-CD11c (301630), BV750 anti-CD19 (302262), allophycocyanin (APC)/Fire 810 anti-CD3 (344858), BV510 anti-IgD (348220), BV605 anti-IgM (314524), phycoerythrin (PE)/Dazzle 594 anti-CXCR5 (356928), APC anti-his (362605), PE/Fire 810 anti-CD27 (302859), APC/Fire 750 anti-CD20 (302358), Zombie NIR (423106), phycoerythrin-indotricarbocyanine (PE-Cy7) anti-

CD71 (334112), BV650 BV570 anti-CD45RO (304226), FITC anti-CCR7 (353216), BV605 anti-HLA-DR (307640), Alexa Fluor 700 anti-CD4 (344622), Alexa Fluor 594 anti-CD8 (301056), peridinin chlorophyll protein Cy5.5 (PerCpCy5.5) (304122), PE/Fire 810 anti-CD27 (302859), BV421 anti-ICOS (313524) (all from Biolegend); Biotin anti-IgG3 (OB9210-08), Alexa Fluor 555 anti-IgG2 (OB907032), PE anti-IgG1 (OB905409), FITC anti-IgA (CBL114FMI), Brilliant Blue 700 (BB700) anti-CD38 (BDB566445), PE-Cy7 anti-PD1 (BDB561272) (all from Fisher).

His-tagged SARS-CoV-2 protein purification. Genes encoding SARS-CoV-2 Wuhan-Hu-1 spike protein (residues 1-1213, GenBank: MN908947.3), the Wuhan-Hu-1 RBD (residues 319-514), BA.1 spike protein were cloned into a pCAGGS mammalian expression vector with a C-terminal hexahistidine tag. Both spike proteins were prefusion stabilized and expression optimized with six proline substitutions (F817P, A892P, A899P, A942P, K986P, V987P), with a disrupted S1/S2 furin cleavage site and a C-terminal foldon trimerization motif (YIPEAPRDGQAYVRKDGEWVLLSTFL) (71). Expi293F cells were transiently transfected, and proteins were recovered via cobalt-charged resin chromatography (G-Biosciences) as previously described (38,39).

HLA class II tetramers. HLA class II tetramers representing the HLA-DPA1*01:03/HLA-DPB1*04:01-restricted SARS-CoV-2 spike protein epitopes $S_{167\text{-}180}$ (TFEYVSQPFLMDLE) and $S_{816\text{-}830}$ (SFIEDLLFNKVTLAD) were constructed by cloning the relevant peptides into an AbVec vector system containing the appropriate HLA alpha and beta chains linked to the peptide of interest via a flexible linker at the N-terminus of the beta chain (51–53). This system includes corresponding leucine zipper motifs on the alpha and beta chains to promote HLA monomer stability. Proteins were expressed in 293F cells transfected with the appropriate AbVec vector. Purified HLA monomer was biotinylated with BioA enzyme in biotinylation buffer (0.2 M NaCl, 0.1 M Tris pH 7.5, 5 mM $MgCl_2$, 5 mM ATP, 0.4 mM Biotin, 5 uM Leupeptin, 1 uM Pepstatin, and 0.2 mM PMSF). The monomer was then tetramerized and fluorochrome labeled by adding Streptavidin-R-Phycoerythrin (Agilent) to the $S_{167\text{-}180}$ monomer or Streptavidin-Allophycocyanin (Agilent) to the $S_{816\text{-}830}$ monomer (72).

Focus reduction neutralization test. Neutralizing antibody titers were performed with authentic SARS-CoV-2 strains and variants as previously described (73).

SUPPLEMENTARY REFERENCES

68. Dati F, Schumann G, Thomas L, Aguzzi F, Baudner S, Bienvenu J, Blaabjerg O, Blirup-Jensen S, Carlström A, Petersen PH, et al. Consensus of a group of professional societies and diagnostic companies on guidelines for interim reference ranges for 14 proteins in serum based on the standardization against the IFCC/BCR/CAP Reference Material (CRM 470). International Federation of Clinical Chemistry. Community Bureau of Reference of the Commission of the European Communities. College of American Pathologists. *European J Clin Chem Clin Biochem J Forum European Clin Chem Soc* (1996) 34:517–20.

69. Bonilla FA, Barlan I, Chapel H, Costa-Carvalho BT, Cunningham-Rundles C, Morena MT de la, Espinosa-Rosales FJ, Hammarström L, Nonoyama S, Quinti I, et al. International Consensus Document (ICON): Common Variable Immunodeficiency Disorders. *J Allergy Clin Immunol Pract* (2016) 4:38–59. doi: 10.1016/j.jaip.2015.07.025

70. Ameratunga R, Woon S-T, Gillis D, Koopmans W, Steele R. New diagnostic criteria for CVID. *Expert Rev Clin Immu* (2014) 10:183–186. doi: 10.1586/1744666x.2014.875274

71. Hsieh C-L, Goldsmith JA, Schaub JM, DiVenere AM, Kuo H-C, Javanmardi K, Le KC, Wrapp D, Lee AG, Liu Y, et al. Structure-based design of prefusion-stabilized SARS-CoV-2 spikes. *Science* (2020) 369:eabd0826. doi: 10.1126/science.abd0826

72. Willis RA, Ramachandiran V, Shires JC, Bai G, Jeter K, Bell DL, Han L, Kazarian T, Ugwu KC, Laur O, et al. Production of Class II MHC Proteins in Lentiviral Vector-Transduced HEK-293T Cells for Tetramer Staining Reagents. *Curr Protoc* (2021) 1:e36. doi: 10.1002/cpz1.36

73. Case JB, Bailey AL, Kim AS, Chen RE, Diamond MS. Growth, detection, quantification, and inactivation of SARS-CoV-2. *Virology* (2020) 548:39–48. doi: 10.1016/j.virol.2020.05.015

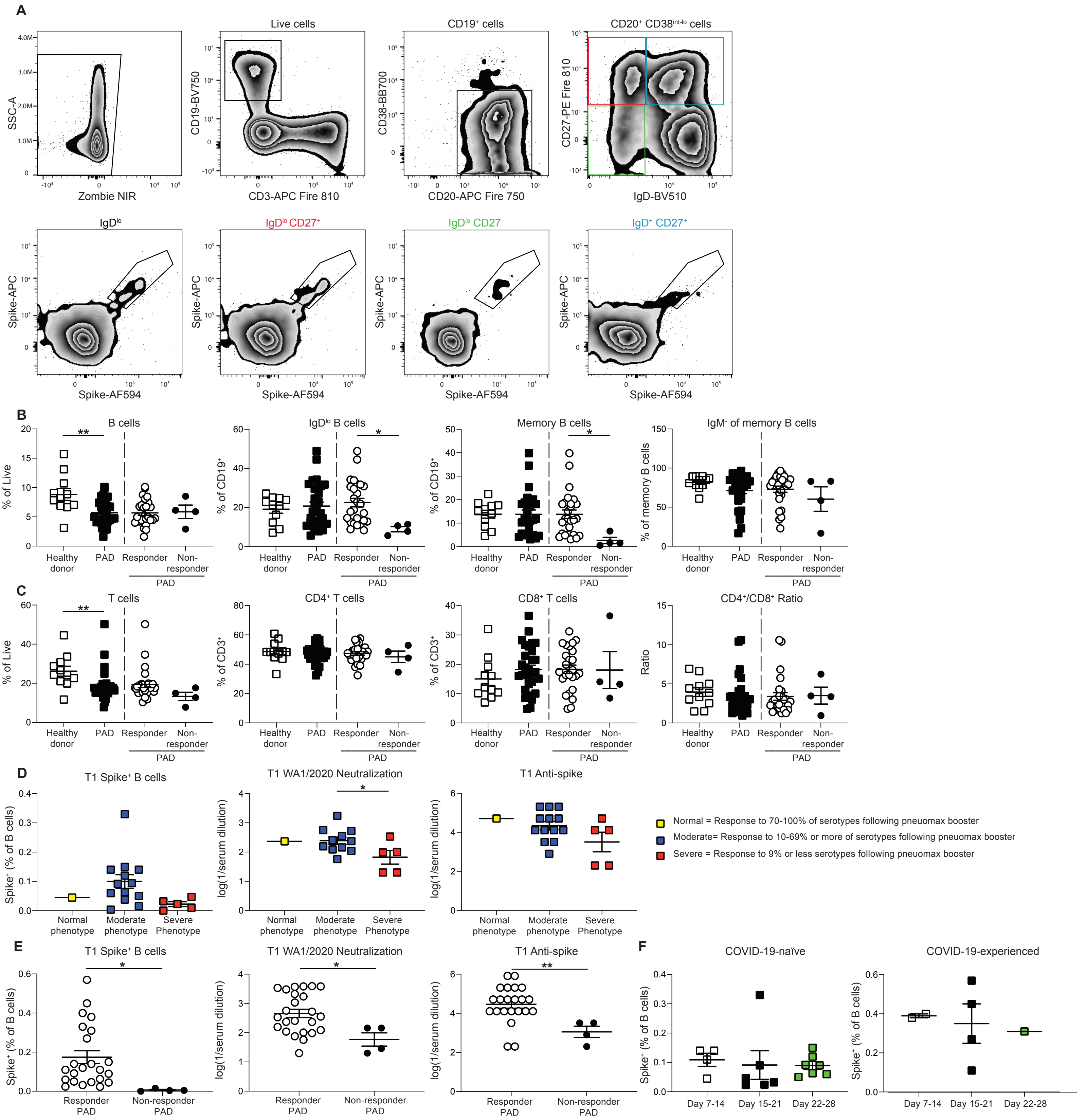
A
Live cells
CD19+ cells
CD20+ CD38int-lo cells
SSC-A
Zombie NIR
CD19-BV750
CD3-APC Fire 810
CD38-BB700
CD20-APC Fire 750
CD27-PE Fire 810
IgD-BV510
IgDlo
IgDlo CD27+
IgDlo CD27-
IgD+ CD27+
Spike-APC
Spike-AF594
B
B cells
IgDlo B cells
Memory B cells
IgM- of memory B cells
% of Live
% of CD19+
% of memory B cells
Healthy donor
PAD
Responder
Non-responder
C
T cells
CD4+ T cells
CD8+ T cells
CD4+/CD8+ Ratio
% of CD3+
Ratio
D
T1 Spike+ B cells
T1 WA1/2020 Neutralization
T1 Anti-spike
Spike+ (% of B cells)
log(1/serum dilution)
Normal phenotype
Moderate phenotype
Severe Phenotype
Normal = Response to 70-100% of serotypes following pneuomax booster
Moderate= Response to 10-69% or more of serotypes following pneuomax booster
Severe = Response to 9% or less serotypes following pneuomax booster
E
Responder PAD
Non-responder PAD
F
COVID-19-naïve
COVID-19-experienced
Day 7-14
Day 15-21
Day 22-28

Figure S1

Figure S1. **Baseline phenotype of PAD patients and healthy donor cohorts**. (**A**) Representative B cell flow cytometry gating scheme. (**B**) Percentage of live cells that are B ($CD19^+CD3^-$) cells at T1 in patient cohorts (left). Percentage of B cells that are IgD^{lo} (middle left) or memory ($IgD^{lo}CD27^+$) at T1 in patient cohorts (middle right). Percentage of memory B cells that are IgM^- in patient cohorts. (right). Non-responders are defined as PAD patients in which the $CD19^+$ IgD^{lo} $Spike^+$ B cells response was <0.02% of total B cells at T1. (**C**) Percentage of live cells that are T ($CD3^+CD19^-$) cells at T1 in patient cohorts. (left) Percentage of T cells that are $CD4^+$ ($CD4^+CD8^-$) (middle left) or $CD8^+$ ($CD8^+CD4^-$) (middle right) in patient cohorts. Ratio of $CD4^+$ to $CD8^+$ T cells (right) in patient cohorts. (**D**) Percentage of IgD^{lo} $Spike^+$ cells among the B (Live $CD19^+$ $CD3^-$) cell population (left), serum neutralizing activity against WA1/2020 (middle), and anti-spike end point antibody titer (right) at T1 in COVID-19-naïve PAD patients with a normal, moderate, or severe immunodeficiency phenotype. Immunodeficiency phenotypes were determined based on the number of protective titers to specific serotypes induced at 4 to 6 weeks following booster vaccination with Pneumovax. (**E**) Percentage of IgD^{lo} $Spike^+$ cells among the B (Live $CD19^+$ $CD3^-$) cell population (left), serum neutralizing activity against WA1/2020 (middle), and anti-spike end point antibody titer (right) at T1 in responder and non-responder PAD patients. (**F**) Percentage of IgD^{lo} $Spike^+$ cells among the B (Live $CD19^+$ $CD3^-$) cell population in COVID-19-naïve PAD (left) and COVID-19-experienced PAD (right) cohorts at T1 in which the T1 PBMC samples were obtained at 7-14 days, 15-21 days, or 22-28 days following administration of the 2nd vaccine dose. Statistical analyses were performed using an unpaired t-test comparing either the healthy and PAD groups or the responder and non-responder groups. Error bars were calculated based on the standard error of the mean. (*, $p < 0.05$; **, $p < 0.01$).

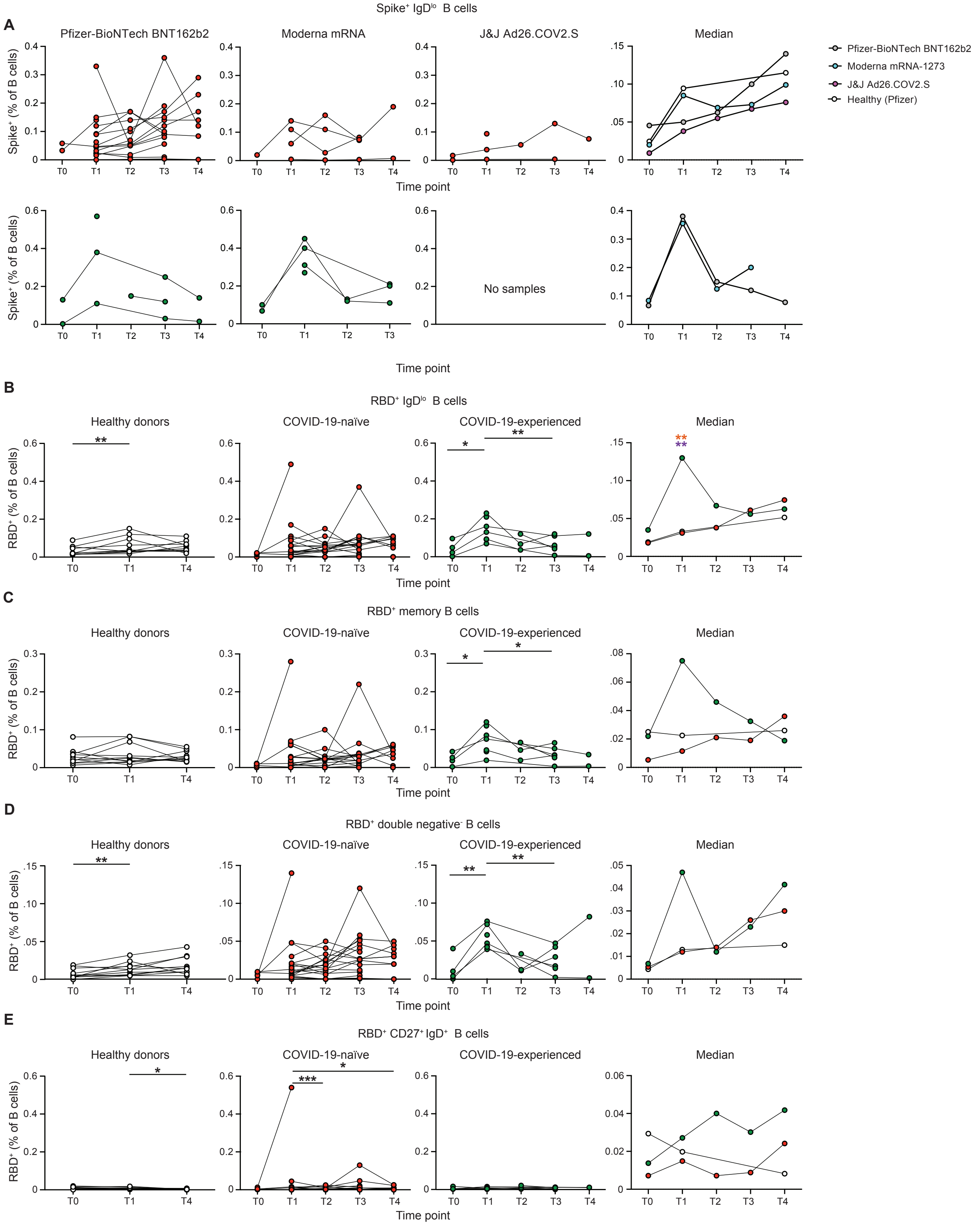
Spike+ IgDlo B cells
A
Pfizer-BioNTech BNT162b2
Moderna mRNA
J&J Ad26.COV2.S
Median
Pfizer-BioNTech BNT162b2
Moderna mRNA-1273
J&J Ad26.COV2.S
Healthy (Pfizer)
Spike+ (% of B cells)
Time point
No samples
B
RBD+ IgDlo B cells
Healthy donors
COVID-19-naïve
COVID-19-experienced
Median
RBD+ (% of B cells)
C
RBD+ memory B cells
D
RBD+ double negative- B cells
E
RBD+ CD27+ IgD+ B cells

Figure S2

Figure S2. **COVID-19-experienced PAD patients have an elevated RBD-specific B cell response following primary vaccination series**. (**A**) Percentage of IgD^{lo} $Spike^{+}$ cells among the B (Live $CD19^{+}$ $CD3^{-}$) cell population in COVID-19-naïve (top) and COVID-19-experienced (bottom) PAD patients that received the Pfizer-BioNTech BNT162b2 (left), Moderna mRNA-1273 (middle left), and J&J Ad26.COV2.S (middle right) vaccines. Median percentage of B cells that comprise each population in all groups is shown on right. There were no COVID-experienced PAD patients that received the J&J Ad26.COV2.S vaccine. Percentage of (**B**) IgD^{lo}, (**C**) memory (IgD^{lo} $CD20^{+}$ $CD38^{int\text{-}lo}$ $CD27^{+}$), (**D**) double negative (IgD^{lo} $CD20^{+}$ $CD38^{int\text{-}lo}$ $CD27^{-}$), and (**E**) IgD^{+} $CD20^{+}$ $CD38^{int\text{-}lo}$ $CD27^{+}$ RBD^{+} cells among the B (Live $CD19^{+}$ $CD3^{-}$) cell population in healthy donors (left, white), COVID-19-naïve PAD (middle left, red), and COVID-19-experienced PAD (middle right, green) cohorts. Median percentage of B cells that comprise each population in all groups is shown on right. Statistical analyses were performed using a mixed effects model (for trends found between time points) or two-way ANOVA (for trends found between groups shown on the median graphs) with Fisher's least significant difference testing. Significance testing between time points was limited to comparisons relative to T1. Above the median graphs, an orange asterisk indicates a comparison between the COVID-19-naïve and COVID-19-experienced groups, and a purple asterisk indicates a comparison between the COVID-19-experienced and healthy donor groups (*, $p < 0.05$; **, $p < 0.01$; ***, $p < 0.001$; ****, $p < 0.001$).

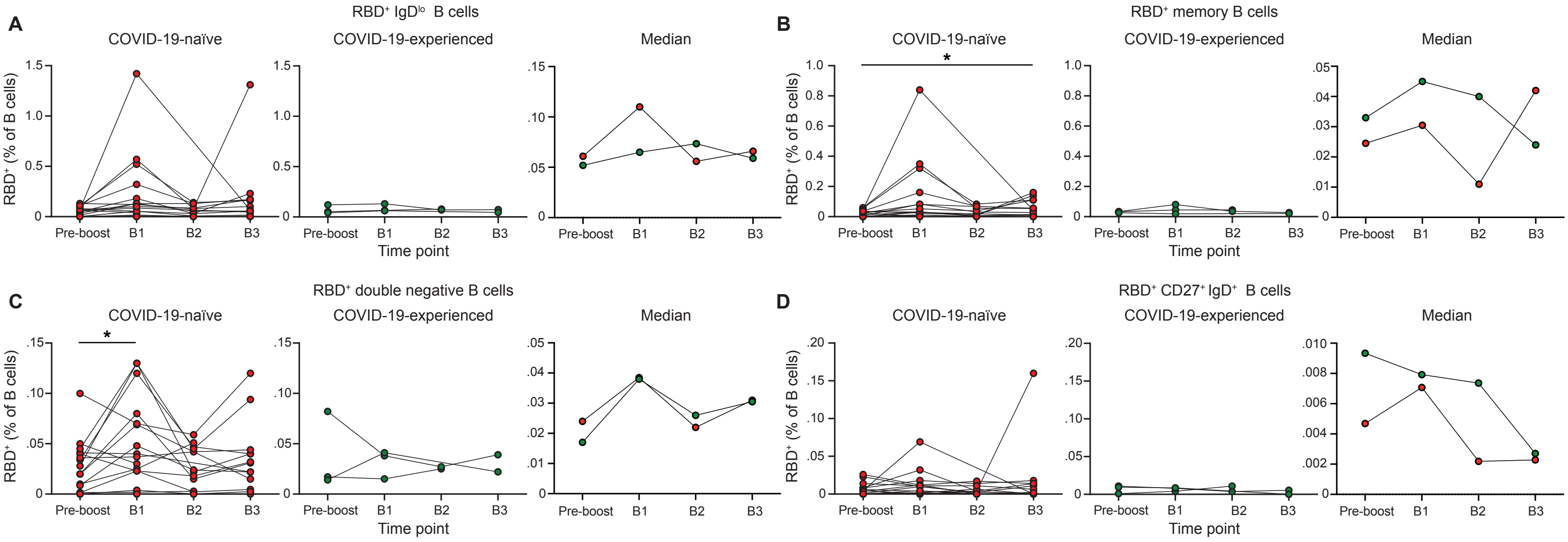

A
RBD+ IgDlo B cells
COVID-19-naïve
COVID-19-experienced
Median
RBD+ (% of B cells)
Pre-boost
B1
B2
B3
Time point
B
RBD+ memory B cells
COVID-19-naïve
COVID-19-experienced
Median
C
RBD+ double negative B cells
COVID-19-naïve
COVID-19-experienced
Median
D
RBD+ CD27+ IgD+ B cells
COVID-19-naïve
COVID-19-experienced
Median

Figure S3

Figure S3. COVID-19-naïve PAD patients have an elevated RBD-specific B cell response following booster vaccination. Percentage of (**A**) IgD^{lo}, (**B**) memory (IgD^{lo} $CD20^{+}$ $CD38^{int-lo}$ $CD27^{+}$), (**C**) double negative (IgD^{lo} $CD20^{+}$ $CD38^{int-lo}$ $CD27^{-}$), and (**D**) IgD^{+} $CD20^{+}$ $CD38^{int-lo}$ $CD27^{+}$ RBD^{+} cells among the B (Live $CD19^{+}$ $CD3^{-}$) cell population in the COVID-19-naïve PAD (left, red) and COVID-19-experienced PAD (middle, green) cohorts. Median percentage of B cells that comprise each population in all groups is shown on right. Pre-boost group consists of the last sample obtained from each patient prior to booster vaccination. Statistical analyses were performed using a mixed effects model (for trends found between time points) with Fisher's least significant difference testing. Significance testing between time points was limited to comparisons relative to pre-boost (*, $p < 0.05$).

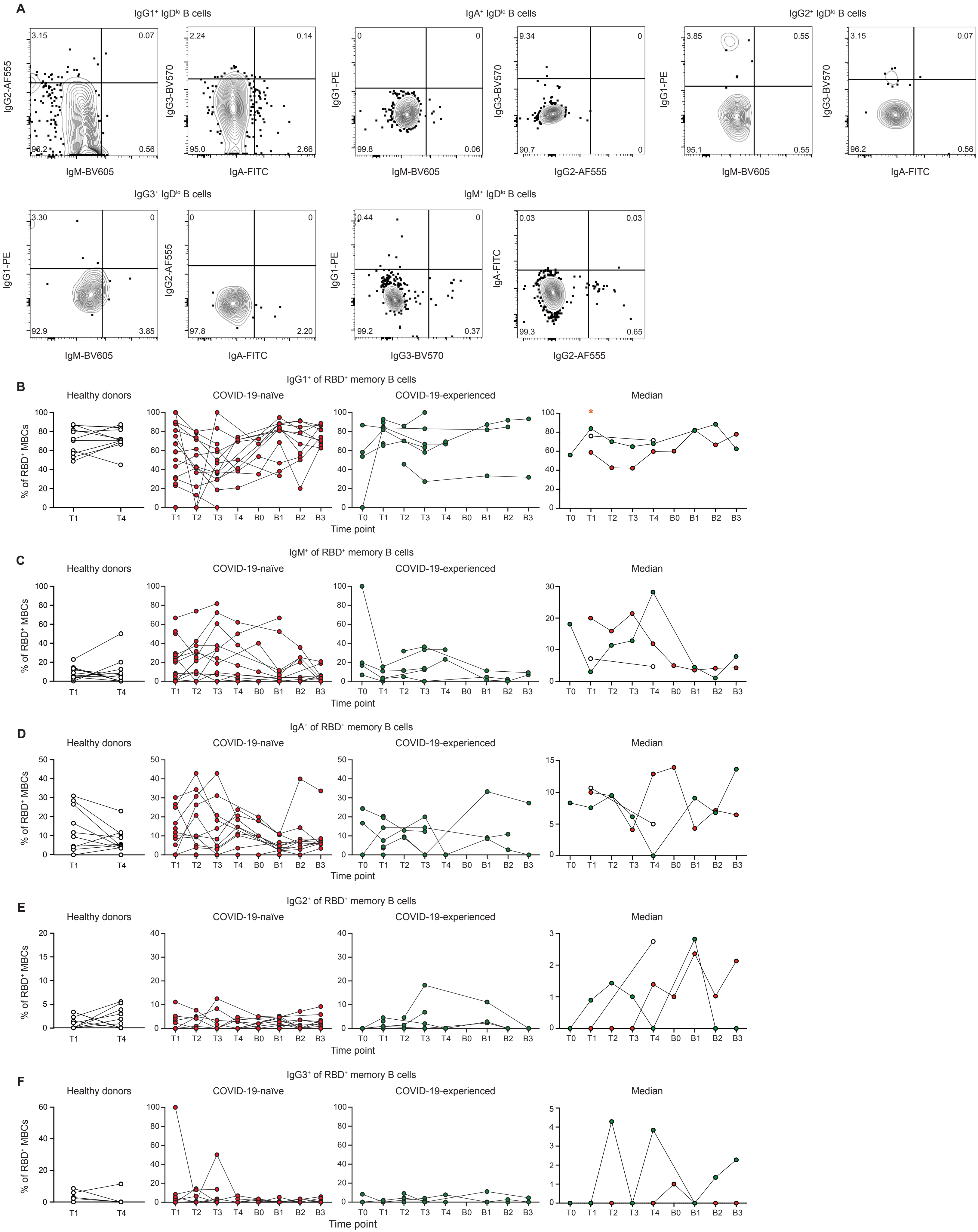
A
IgG1⁺ IgDˡᵒ B cells
IgA⁺ IgDˡᵒ B cells
IgG2⁺ IgDˡᵒ B cells
IgG3⁺ IgDˡᵒ B cells
IgM⁺ IgDˡᵒ B cells
IgG2-AF555
IgG3-BV570
IgG1-PE
IgM-BV605
IgA-FITC
IgG3-BV570
IgG2-AF555
B
IgG1⁺ of RBD⁺ memory B cells
C
IgM⁺ of RBD⁺ memory B cells
D
IgA⁺ of RBD⁺ memory B cells
E
IgG2⁺ of RBD⁺ memory B cells
F
IgG3⁺ of RBD⁺ memory B cells
Healthy donors
COVID-19-naïve
COVID-19-experienced
Median
% of RBD⁺ MBCs
Time point

Figure S4

Figure S4. Isotype composition of SARS-CoV-2 RBD-specific memory B cell response following vaccination in PAD patients. (**A**) Representative flow cytometry plots of the expression of other isotypes in $IgG1^+$, IgA^+, $IgG2^+$, $IgG3^+$, and IgM^+ IgD^{lo} B cells. Percentage of RBD^+ memory B cells that are (**B)** $IgG1^+$, (**C**) IgM^+, (**D**) IgA^+, (**E**) $IgG2^+$, and (**F**) $IgG3^+$ in healthy donors (left, white), COVID-19-naïve PAD (middle left, red), and COVID-19-experienced PAD (middle right, green) cohorts. Median percentage of B cells that comprise each population in all groups is shown on right. Statistical analyses were performed using a two-way ANOVA (for trends found between groups shown on the median graphs) with Fisher's least significant difference testing. Above the median graphs, an orange asterisk indicates a comparison between the COVID-19-naïve and -experienced groups (*, $p < 0.05$).

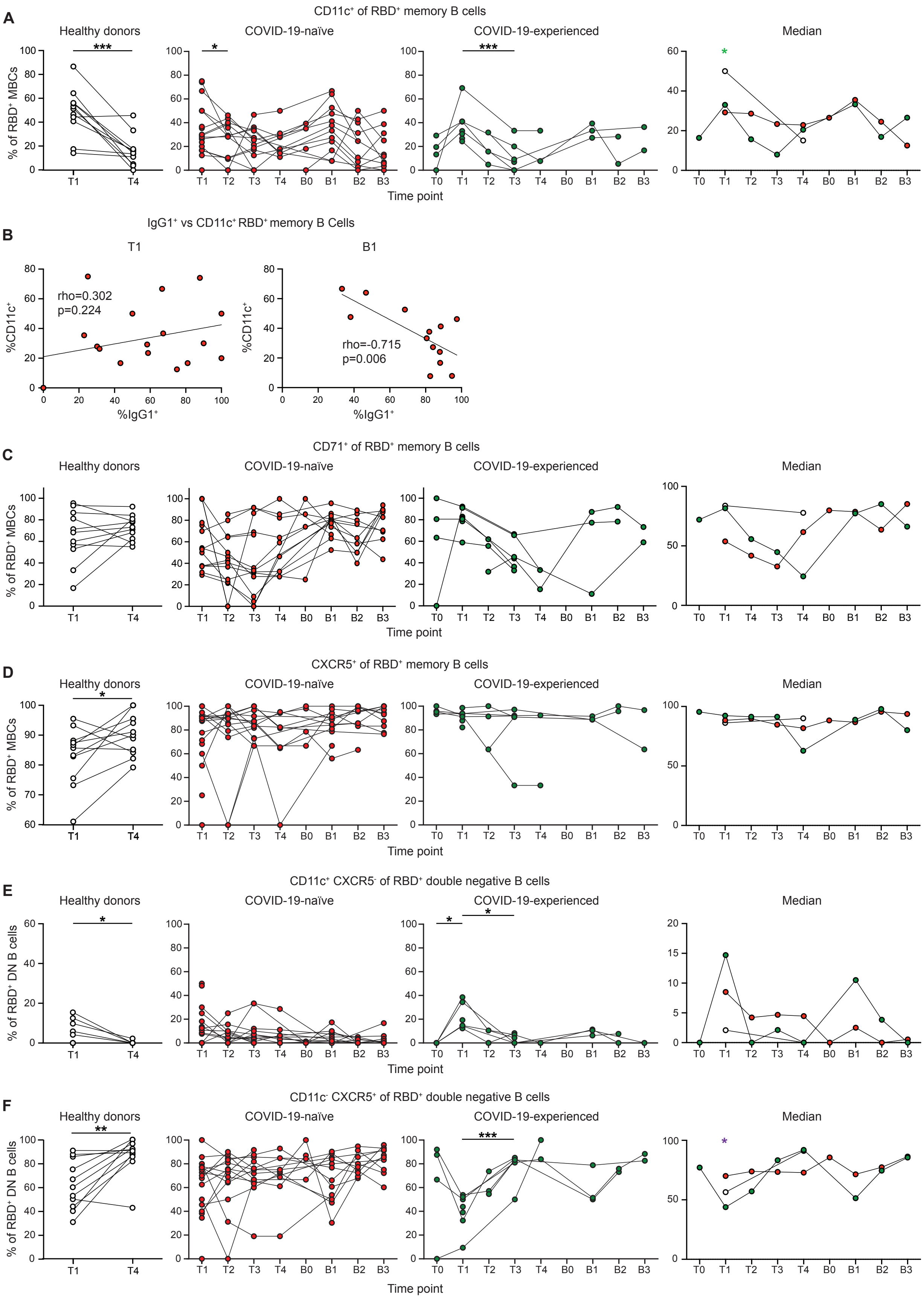
A
CD11c⁺ of RBD⁺ memory B cells
Healthy donors
COVID-19-naïve
COVID-19-experienced
Median
% of RBD⁺ MBCs
Time point
B
IgG1⁺ vs CD11c⁺ RBD⁺ memory B Cells
T1
B1
rho=0.302
p=0.224
rho=-0.715
p=0.006
%CD11c⁺
%IgG1⁺
C
CD71⁺ of RBD⁺ memory B cells
Healthy donors
COVID-19-naïve
COVID-19-experienced
Median
% of RBD⁺ MBCs
Time point
D
CXCR5⁺ of RBD⁺ memory B cells
Healthy donors
COVID-19-naïve
COVID-19-experienced
Median
% of RBD⁺ MBCs
Time point
E
CD11c⁺ CXCR5⁻ of RBD⁺ double negative B cells
Healthy donors
COVID-19-naïve
COVID-19-experienced
Median
% of RBD⁺ DN B cells
Time point
F
CD11c⁻ CXCR5⁺ of RBD⁺ double negative B cells
Healthy donors
COVID-19-naïve
COVID-19-experienced
Median
% of RBD⁺ DN B cells
Time point

Figure S5

Figure S5. RBD-specific memory B cells from PAD patients display reduced CD11c expression class. (**A**) Percentage of RBD^+ memory B cells that are $CD11c^+$ in healthy donors (left, white), COVID-19-naïve PAD (middle left, red), and COVID-19-experienced PAD (middle right, green) cohorts. Median percentage of $CD11c^+$ cells in all groups is shown on right. (**B**) Correlation between percentage of RBD^+ memory B cells that are $IgG1^+$ and $CD11c^+$ at T1 (left) or B1 (right). Associations for **B** are calculated using Pearson rank correlation and shown with Pearson trend lines for visualization. Percentage RBD^+ memory B cells that are (**C**) $CD71^+$ or (**D**) $CXCR5^+$ in healthy donors (left, white), COVID-19-naïve PAD (middle left, red), and COVID-19-experienced PAD (middle right, green) cohorts. Median percentage of B cells that comprise each population in all groups is shown on right. Percentage of RBD^+ double negative B cells that are (**E**) $CD11c^+$ $CXCR5^-$ or (**F**) $CD11c^-$ $CXCR5^+$ in healthy donors (left, white), COVID-19-naïve PAD (middle left, red), and COVID-19-experienced PAD (middle right, green) cohorts. Median percentage of double negative B cells that comprise each population in all groups is shown on right. Statistical analyses in **A, C-F** were performed using a mixed effects model (for trends found between time points) or a two-way ANOVA (for trends found between groups shown on the median graphs) with Fisher's least significant difference testing. Significance testing between time points was limited to comparisons relative to T1. Above the median graphs, a green asterisk indicates a comparison between the COVID-19-naïve and healthy donor groups, a purple asterisk indicates a comparison between the COVID-19-experienced and healthy donor groups (*, $p < 0.05$; **, $p < 0.01$; ***, $p < 0.001$).

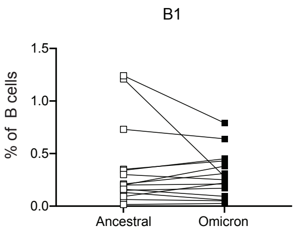

Figure S6

Figure S6. Booster vaccination induces similar percentages of Ancestral and Omicron Spike^{+} B cells. Paired comparison of percentage of IgD^{lo} ancestral Spike^{+} and Omicron^{+} cells among the B (Live CD19^{+} CD3^{-}) cell population at B1.

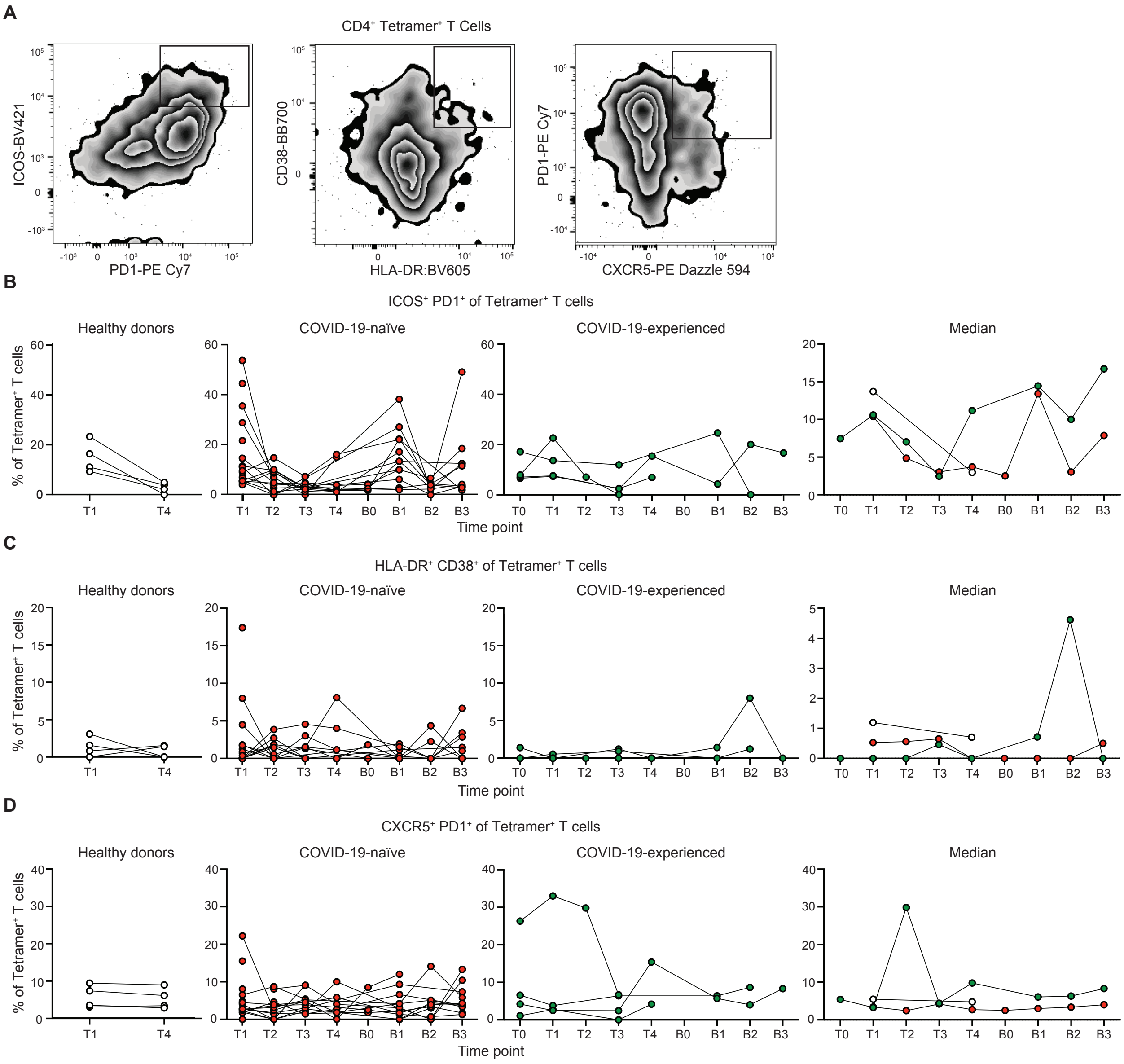
A
CD4⁺ Tetramer⁺ T Cells
ICOS-BV421
PD1-PE Cy7
CD38-BB700
HLA-DR:BV605
PD1-PE Cy7
CXCR5-PE Dazzle 594
B
ICOS⁺ PD1⁺ of Tetramer⁺ T cells
Healthy donors
COVID-19-naïve
COVID-19-experienced
Median
% of Tetramer⁺ T cells
T0 T1 T2 T3 T4 B0 B1 B2 B3
Time point
C
HLA-DR⁺ CD38⁺ of Tetramer⁺ T cells
Healthy donors
COVID-19-naïve
COVID-19-experienced
Median
% of Tetramer⁺ T cells
Time point
D
CXCR5⁺ PD1⁺ of Tetramer⁺ T cells
Healthy donors
COVID-19-naïve
COVID-19-experienced
Median
% of Tetramer⁺ T cells
Time point

Figure S7

Figure S7. Additional phenotyping of SARS-CoV-2-specific CD4$^+$ T cell response following vaccination in PAD patients. (**A**) Representative flow cytometry plots of the expression of PD1, ICOS, CD38, HLA-DR, and CXCR5 on Tetramer$^+$ CD4$^+$ (Live CD3$^+$ CD19$^-$ CD4$^+$ CD8$^-$ $S_{167-180}{}^+$ or $S_{816-830}{}^+$) T cells. Percentage of Tetramer$^+$ CD4$^+$ T cells that are (**B**) ICOS$^+$PD1$^+$, (**C**) HLA-DR$^+$CD38$^+$, and (**D**) CXCR5$^+$PD1$^+$ in healthy donors (left, white), COVID-19-naïve PAD (middle left, red), and COVID-19-experienced PAD (middle right, green) cohorts. Median percentage of Tetramer$^+$ T cells that comprise each population in all groups is shown on right.

Patient	Age	Sex	Vaccine Type	Booster Type	Diagnosis	COVID-19 infection to 1st vaccine (days)
101	59	F	Pfizer	Pfizer	CVID	--
102	25	F	Pfizer	Pfizer	CVID	--
103	56	F	Pfizer	--	CVID	--
104	41	F	Pfizer	Pfizer	CVID	--
105	30	M	Pfizer	Pfizer	CVID	--
106	61	F	Pfizer	--	CVID	--
107	73	F	Pfizer	Pfizer	Hypogam	96
108	61	F	Pfizer	Pfizer	CVID	--
109	37	F	J&J	Pfizer	SAD	--
110	46	F	Pfizer	Pfizer	SAD	--
111	59	F	Pfizer	Pfizer	Hypogam	--
112	44	M	Moderna	Moderna	CVID	--
114	34	F	Pfizer	--	SAD	90
115	20	F	Moderna	--	CVID	181
116	26	F	J&J	--	SAD	--
117	82	F	Moderna	--	CVID	--
118	61	F	Moderna	--	CVID	--
119	21	F	Pfizer	Pfizer	Hypogam	276
120	41	F	Moderna	Moderna	CVID	--
121	70	F	Pfizer	Pfizer	CVID	--
122	49	F	Pfizer	--	CVID	117
123	70	F	Pfizer	Pfizer	Hypogam	--
124	54	F	Moderna	--	CVID	222
125	56	F	Moderna	--	Hypogam	106
126	57	M	J&J	Pfizer	CVID	--
127	56	F	Pfizer	Pfizer	CVID	--
128	63	F	Pfizer	Pfizer	SAD	--
129	37	F	Pfizer	Pfizer	CVID	--
130	48	F	Moderna	Moderna	SAD	144
131	29	F	Pfizer	--	CVID	36
368-05	36	M	Pfizer	--	Healthy donor	--
368-17	37	M	Pfizer	--	Healthy donor	--
368-24	55	F	Pfizer	--	Healthy donor	--
368-25	45	F	Pfizer	--	Healthy donor	--
368-27	47	M	Pfizer	--	Healthy donor	--
368-29	30	F	Pfizer	--	Healthy donor	--
368-34	28	M	Pfizer	--	Healthy donor	--
368-36	48	F	Pfizer	--	Healthy donor	--
368-37	44	M	Pfizer	--	Healthy donor	--
368-38	42	F	Pfizer	--	Healthy donor	--
368-40	33	F	Pfizer	--	Healthy donor	--

Table S1. Characteristics of patient cohort

F, female; M, male; CVID, common variable immune defeiciency; hypogam, hypogammaglobulinemia; SAD, specific antibody deficiency disorder.

Patient	B cells (% of Live)	IgD^{lo} (% of CD19^{+})	IgD^{lo} CD27^{+} (% of CD19^{+})	CD3^{+} T cells (% of Live)	CD4^{+} T cells (% of CD3^{+})	CD8^{+} T cells (% of CD3^{+})	CD4^{+}/CD8^{+} Ratio
101	4.07	34.3	18.8	34.6	57.6	10	5.76
102	5.21	12.5	5.16	28.3	47.4	28.2	1.68
103	4.74	11.7	4.61	50.2	51.4	24.5	2.10
104*	2.9	7.81	0.53	7.72	44.2	13.2	3.35
105	4.21	11.4	5.51	10.3	32.4	26.1	1.24
106*	6.13	5.63	1.68	12.7	52.9	8.48	6.24
107	3.02	27.1	15.1	17.1	42.9	18.9	2.27
108	4.42	30.6	17.1	11.8	45.7	15.1	3.03
109	9.89	12.8	8.27	16.5	48.6	16.9	2.88
110	3.61	32	18.1	18.1	49.3	18	2.74
111	4.01	44.5	34.5	15.2	47.8	16	2.99
112*	8.5	10.7	1.53	14.9	34.6	36.5	0.95
114	6.65	30.9	20.1	24.9	56.4	16.3	3.46
115	6.78	24.6	18.1	15	43.6	22.9	1.90
116	1.61	18.8	11.9	15.7	38.1	27.3	1.40
117	4.45	14.5	4.97	17.2	50.1	4.8	10.4
118	4.67	14.7	10.3	17.6	46.5	15.6	2.98
119	4.95	22.8	11.9	15.3	47.5	17.4	2.73
120	6.99	31.7	11.9	17.6	46.1	11.3	4.08
121	7.43	21.8	18.2	19.6	54.9	8.01	6.85
122	6.54	48.9	11	18.6	39.9	9.3	4.29
123	8.75	10.9	39.8	17.6	55	5.18	10.6
124	8.08	17.9	4.09	17.2	48.6	21.5	2.26
125	10.1	13.3	11.3	19.9	50.3	21.5	2.34
126*	5.89	11.4	6.62	17.7	48.5	14	3.46
127	4.92	17.1	4.82	19.6	56.7	16.4	3.46
128	2.77	29.5	3.09	16.3	44.9	19.9	2.26
129	8.45	8.89	20.8	17.5	44.1	26.6	1.66
130	6.74	33.9	3.54	18.4	46	27.2	1.69
131	4.43	8.39	25.3	12.7	39.1	31.2	1.25
368-05	10.7	12.3	8.52	30.8	47.3	32	1.48
368-17	8.85	25.1	15.1	19.9	57.5	8.68	6.62
368-24	7.82	16.3	11.8	28.7	60.8	12.1	5.02
368-25	13.1	22.9	17.3	21	48.9	10.4	4.70
368-27	3.12	24.8	22.4	44.5	33.3	22.3	1.49
368-29	8.19	21.3	14.1	26.6	48.3	19.2	2.52
368-34	15.7	8.93	6.25	33.6	43.7	11.3	3.87
368-36	6.64	20.8	16	24.2	54	16.3	3.31
368-37	6.88	23.9	18.5	22.6	48.4	6.98	6.93
368-38	7.05	7.13	4.5	11.7	44.7	10.1	4.43
368-40	9.09	27.1	17.6	24.3	48.2	16	3.01

Table S2. Cellular phenotype of patient cohort

* Patients classified as non-responders (IgD^{lo} Spike^{+} B cell response <0.02% of total B cells at day 7 to 28 post vaccination).

Patient	Most Recent CBC		Lymphocyte Subpopulation at the Time of Diagnosis					Immunoclobulin levels at the Time of Diagnosis			Most Recent IgG***
	WBC	ALC	CD3	CD4	CD8	CD19	CD16/56	IgG	IgA	IgM	
**Normal range**	**3.8 - 9.9 (1000s per mm^3)**	**1,000 - 4,800 (per mm^3)**	**661 - 1963 (per mm^3)**	**490 - 1294 (per mm^3)**	**187 - 781 (per mm^3)**	**110 - 488 (per mm^3)**	**76 - 467 (per mm^3)**	**700 - 1600 mg/dL**	**70 - 400 mg/dL**	**40 - 230 mg/dL**	**mg/dL**
101	5.8	2400	2118	1668	470	283	258	477	53	62	1065
102	4	1000	895	426	432	227	127	560	62	24	1134
103	4.5	1674	1450	934	456	187	180	535	140	100	828
104*	5.1	1000	818	601	193	251	18	<40	<4	<5	725
105	12	1500	974	384	487	280	221	<300	<10	<25	947
106*	7.2	1600	--	--	--	--	--	432	89	95	830
107	6.5	1900	1109	775	294	80	88	514	72	165	505
108	9.5	3500	Reported normal in immunology clinic note					529	188	35	938
109	6.4	1100	--	--	--	--	--	895	142	105	1345
110	4.8	1700	1632	859	738	184	59	888	73	37	1462
111	5.2	860	--	--	--	--	--	465	151	98	836
112*	4.3	1000	1162	375	663	134	151	low**	<6	<4	1303
114	8.3	1900	1702	1054	547	243	41	911	221	58	1291
115	15.6	2400	--	--	--	--	--	340	12	reported nl	1261
116	8	2400	1974	1167	690	262	71	983	162	52	983
117	5.4	1600	2194	1646	549	462	144	553	175	49	1143
118	7.3	1700	Reported normal in immunology clinic note					213	14	14	758
119	5	2200	2055	1396	790	211	79	473	120	167	877
120	8.3	2600	1848	1282	501	157	199	528	136	155	1164
121	6.2	2400	859	716	119	95	227	488	121	131	989
122	10.6	2720	3300	2274	1026	580	446	606	204	30	743
123	8	1500	1055	835	205	315	158	645	162	45	887
124	9.1	1190	Reported low NK cells only in immunology clinic note					502	42	95	547
125	4.9	2000	1837	1086	725	475	308	387	255	46	657
126*	4.5	1600	Reported low CD19 at 39, all others normal in immunology note					177	66	<25	860
127	3.8	1400	925	638	287	64	43	516	<25	521	781
128	7.2	800	655	389	236	209	78	629	196	80	990
129	5	1420	--	--	--	--	--	261	<10	<25	1240
130	6.27	1610	--	--	--	--	--	814	99	198	1145
131	10.79	1700	--	--	--	--	--	346	<10	<25	1036

Table S3. PAD patient laboratory values

CBC - complete blood count; WBC - white blood count, ALC - absolute lymphocyte count; Ig - immunoglobulin; dL - deciliter. Empty cells (dashed lines) indicate that the test was not performed.

* Patients classified as non-responders (IgD^{lo} $Spike^{+}$ B cell response <0.02% of total B cells at day 7 to 28 post vaccination).

** Pre-IVIG IgG titer was recorded as low in records

*** Most recent IgG taken while patients are receiving immunoglobulin replacement therapy

-- Data unavailable

Patient	*S. pneumoniae* titer**	Tetanus titer IU/ml****	Diphtheria titer IU/ml****
101	8/23	N/A	--
102	7/23	0.51	--
103	10/23	Positive	Positive
104*	0/23	Positive	--
105	0/23***	Negative	--
106*	4/13	4.59	--
107	4/23	0.97	0.09
108	2/23	0.06	0.01
109	6/23	>7	2.34
110	5/23	N/A	--
111	11/14	0.98	--
112*	--	N/A	--
114	12/23	1.56	--
115	Unprotective	--	--
116	14/23	0.36	Positive
117	11/23	0.01	--
118	--	--	--
119	19/23	0.45	--
120	14/23	>2.24	--
121	8/23	0.46	--
122	10/23	0.72	--
123	20/23	>2.24	--
124	2/14	--	--
125	16/23	2.2	--
126*	3/23***	0.24	Undetectable
127	0/23	--	--
128	14/23	0.87	0.07
129	0/23	--	--
130	0/23	2.75	--
131	--	1.73	0.35

Table S4. PAD patient responses to other vaccine antigens at the time of diagnosis

IU - international unit. N/A - data was obtained but not available in the medical record. Dashed lines indicate that the test was not performed.

* Patients classified as non-responders (IgD^{lo} $Spike^{+}$ B cell response <0.02% of total B cells at day 7 to 28 post vaccination).
** Indicates the number of tested anti-*Streptococcus pneumoniae* serotypes with a level above 1.3 μg/ml
4-6 weeks following pneumovax booster vaccination.
*** Pre-pneumovax booster values; patient started on immunoglobulin replacement prior to post-booster recheck.
**** A value of ≥ 0.01 IU/ml is considered positive. In some records, a positive notation was indicated but no value was provided.
-- Data unavailable
